# Comprehensive Analyses of the Immunological and Prognostic Roles of an IQGAP3AR/let-7c-5p/IQGAP3 Axis in Different Types of Human Cancer

**DOI:** 10.3389/fmolb.2022.763248

**Published:** 2022-02-22

**Authors:** Yixiao Yuan, Xiulin Jiang, Lin Tang, Hong Yang, Juan Wang, Dahang Zhang, Lincan Duan

**Affiliations:** ^1^ Department of Thoracic Surgery, The Third Affiliated Hospital of Kunming Medical University, Kunming, China; ^2^ Key Laboratory of Animal Models and Human Disease Mechanisms of Chinese Academy of Sciences and Yunnan Province, Kunming Institute of Zoology, Kunming, China; ^3^ Department of Urology, The Third Affiliated Hospital of Kunming Medical University, Kunming, China

**Keywords:** IQGAP3, let-7c-5p, human cancer, prognosis, immunotherapy

## Abstract

IQ motif containing GTPase-activating protein 3 (IQGAP3) is a member of the Rho family of guanosine-5′-triphosphatases (GTPases). IQGAP3 plays a crucial part in the development and progression of several types of cancer. However, the prognostic, upstream-regulatory, and immunological roles of IQGAP3 in human cancer types are not known. We found that IQGAP3 expression was increased in different types of human cancer. The high expression of IQGAP3 was correlated with tumor stage, lymph node metastasis, and a poor prognosis in diverse types of human cancer. The DNA methylation of IQGAP3 was highly and negatively correlated with IQGAP3 expression in diverse cancer types. High DNA methylation in IQGAP3 was correlated with better overall survival in human cancer types. High mRNA expression of IQGAP3 was associated with tumor mutational burden, microsatellite instability, immune cell infiltration, and immune modulators. Analyses of signaling pathway enrichment showed that IQGAP3 was involved in the cell cycle. IQGAP3 expression was associated with sensitivity to a wide array of drugs in cancer cells lines. We revealed that polypyrimidine tract–binding protein 1 (PTBP1) and an IQGAP3-associated lncRNA (IQGAP3AR)/let-7c-5p axis were potential regulations for IQGAP3 expression. We provided the first evidence to show that an IQGAP3AR/let-7c-5p/IQGAP3 axis has indispensable roles in the progression and immune response in different types of human cancer.

## Introduction

Cancer is a major cause of death worldwide and results in considerable social and economic burdens. Despite improvements in the diagnosis and treatment of cancer, the prevalence of cure is low (Siegel et al., 2019). Therefore, the identification of specific and sensitive biomarkers for the diagnosis and treatment of cancer is very important.

IQ motifs containing GTPase-activating protein 3 (IQGAP3) is a member of the Rho family of guanosine-5′-triphosphatase (GTPase). Of the proteins IQGAP1, IQGAP2, and IQGAP3 (Swart-Mataraza et al., 2002), IQGAP1 has been shown to participate mainly in the regulation of cellular motility (Schmidt et al., 2008). Studies have revealed that IQGAP1 promotes the intrinsic GTPase activity of Cdc42, thereby resulting in altered cellular morphology (Swart-Mataraza et al., 2002). IQGAP2 has been reported to be a tumor-suppressor gene in different types of cancer (Swart-Mataraza et al., 2002). IQGAP3 was identified as a member of the IQGAP family in 2007 (Wang et al., 2007) and has indispensable roles in neuronal morphogenesis (Wang et al., 2007). Studies have indicated that IQGAP3 is located mainly in chromosome 1 at 1q21.3 and has been reported to act as an oncogene in different types of human cancer (Xu et al., 2016; Dongol et al., 2020; Zeng et al., 2020). For instance, IQGAP3 expression has been shown to be upregulated in high-grade serous ovarian cancer, and IQGAP3 depletion inhibits the proliferation, migration, and invasion of ovarian cancer cell lines markedly (Dongol et al., 2020). IQGAP3 silencing has been demonstrated to significantly reduce the proliferation, migration, and invasion ability, and induce apoptosis in pancreatic cancer cell lines (Xu et al., 2016). Zhang et al. found that IQGAP3 had high expression in breast cancer tissues and cell lines, and its high expression was correlated with the clinical stage, tumor node metastasis stage, and a poor prognosis (Hua et al., 2020). High expression of IQGAP3 has been observed in colorectal cancer (Wu et al., 2019), hepatocellular carcinoma (Shi et al., 2017), bladder cancer (Xu et al., 2019), and gastric cancer (Huang et al., 2021). Hence, IQGAP3 appears to have important roles in cancer progression and could be a promising biomarker. However, the prognostic and immunological roles of IQGAP3 in human cancer are not known.

In the present study, we first employed public databases to analyze the expression and prognosis in different types of human cancer. Our results indicated that IQGAP3 expression was upregulated significantly in bladder urothelial carcinoma (BLCA), breast-invasive carcinoma (BRCA), cervical squamous cell carcinoma and endocervical adenocarcinoma (CESC), cholangiocarcinoma (CHOL), colon adenocarcinoma (COAD), esophageal carcinoma (ESCA), glioblastoma multiforme (GBM), head and neck squamous cell carcinoma (HNSC), kidney renal clear cell carcinoma (KIRC), kidney renal papillary cell carcinoma (KIRP), lung adenocarcinoma (LUAD), lung squamous cell carcinoma (LUSC), pancreatic adenocarcinoma (PAAD), pheochromocytoma and paraganglioma (PCPG), prostate adenocarcinoma (PRAD), rectal adenocarcinoma (READ), stomach adenocarcinoma (STAD), thyroid carcinoma (THCA), and uterine corpus endometrial carcinoma (UCEC). High expression of IQGAP3 was associated with poor overall survival (OS) in adrenocortical carcinoma (ACC), KIRC, KIRP, acute myeloid leukemia (LAML), brain lower-grade glioma (LGG), liver hepatocellular carcinoma (LIHC), LUAD, mesothelioma (MESO), and uveal melanoma (UVM). High expression of IQGAP3 was also associated with short disease-free survival (DFS) in ACC, chromophobe kidney cancer (KICH), KIRP, LGG, LIHC, MESO, PRAD, skin cutaneous melanoma (SKCM), and UVM. IQGAP3 expression was not only related to the tumor stage of ACC, BRCA, KICH, KIRC, KIRP, LIHC, LUAD, LUSC, ovarian serous cystadenocarcinoma (OV), PAAD, and THCA, but also correlated with lymph node metastasis in BLCA, BRCA, CESC, CHOL, COAD, ESCA, HNSC, KICH, KIRC, KIRP, LIHC, LUAD, LUSC, MESC, PAAD, PRAD, READ, STAD, and THCA. In addition, the low level of DNA methylation and high copy-number variation (CNV) of IQGAP3 significantly affected its expression in different types of cancer. IQGAP3 expression was closely associated with tumor mutational burden (TMB), microsatellite instability (MSI), infiltration of immune cells, and immune modulators.

In addition, we identified a 3060-base pair (bp) long non-coding (lnc) RNA, termed “IQGAP3AR” (IQGAP3-associated lncRNA: ENSG00000234072), which showed high expression in human cancer that predicted a poor prognosis. We also found that the transcription factor PTBP1 and IQGAP3AR/let-7c-5p axis were potential regulators of IQGAP3 expression. High expression of IQGAP3AR correlated with the tumor stage and poor prognosis in different types of cancer. Let-7c-5p expression was decreased in CHOL, BRCA, BLCA, UCEC, THCA, SATD, LUSC, LUAD, LIHC, KICH, HNSC, and COAD. High expression of let-7c-5p was correlated with a good prognosis in BRCA, CECS, ESCA, HNSC, KIRP, LIHC, LUAD, and LUSC. We also showed that IQGAP3 expression was positively correlated with sensitivity to different types of drugs in the Genomics of Drug Sensitivity in Cancer (GDSC) database. Finally, we undertook real-time reverse transcription-quantitative polymerase chain reaction (RT-qPCR) and immunohistochemistry (IHC) assays to show that IQGAP3 had high expression in non–small-cell lung cancer (NSCLC) cell lines and cancer tissue. We provided, for the first time, evidence that the IQGAP3AR/let-7c-5p/IQGAP3 axis has indispensable roles in the progression and immune response in different types of human cancer.

## Materials and Methods

### Analysis of the Expression of IQGAP3 in Pan-Cancer

We employed the TIMER (https://cistrome.shinyapps.io/timer/) (Li et al., 2017), Oncomine (https://www.oncomine.org), and GEPIA databases (http://gepia.cancer-pku.cn/) (Tang et al., 2017) to analyze the expression of IQGAP3 in pan-cancer, and the CCLE tools (https://portals.broadinstitute.org/ccle/) (Ghandi et al., 2019) were employed to examine the expression of IQGAP3 in diverse cancer cells lines. UALCAN tools (http://ualcan.path.uab.edu/) (Chandrashekar et al., 2017) were used to analyze the protein of IQGAP3 in different cancers. The expression of let-7c-5p was analyzed by using starBase (Li et al., 2014). The Kaplan–Meier plotter (http://kmplot.com/analysis/) (Hou et al., 2017) was employed to examine the prognosis of let-7c-5p in pan-cancer.

### Analysis of the Prognosis and Clinical Information of IQGAP3 in Pan-Cancer

We employed the GEPIA (http://gepia.cancer-pku.cn/) and prognostic databases (http://dna00.bio.kyutech.ac.jp/PrognoScan/index.html) (Tang et al., 2017; Mizuno et al., 2009) to analyze the OS and RFS of IQGAP3 in pan-cancer; additionally, the correlation between the tumor stage and IQGAP3 expression was analyzed by using GEPIA. Tumor stage, lymph node metastasis, and expression of let-7c-5p were analyzed by using UALCAN tools (http://ualcan.path.uab.edu/) (Chandrashekar et al., 2017). We also employed the prognosis tools to verify the prognosis of IQGAP3 in pan-cancer.

### Analysis of the DNA Methylation and Gene Mutation of IQGAP3 in Pan-Cancer

The DNA methylation of IQGAP3 was analyzed by Ualcan tools (http://ualcan.path.uab.edu/) (Chandrashekar et al., 2017), the correlation between the OS and DNA methylation level was analyzed by the Methsurv database (https://biit.cs.ut.ee/methsurv/) (Modhukur et al., 2018). The mutation information of IQGAP3 in pan-cancer was analyzed by the cbioportal database (https://www.cbioportal.org/) (Cerami et al., 2012).

### Starbase Database

We employed the starBase database (http://starbase.sysu.edu.cn/) to forecast the potential miRNAs of IQGAP3 (Li et al., 2014), and examine the expression, prognosis, and correlation between let-7c-5p and IQGAP3, we also used the starbase to predict the binding with between the miRNA, mRNA, and lncRNA.

### Analysis of the Function of IQGAP3 in Pan-Cancer

We employed the CancerSEA database (http://biocc.hrbmu.edu.cn/CancerSEA/) analysis the function of IQGAP3 in pan-cancer (Yuan et al., 2019), the LinkedOmics (http://www.linkedomics.org/admin.php) was employed to analyze the KEGG pathway of IQGAP3 in LUAD (Vasaikar et al., 2018).

### Analysis of the Gene and Protein That Interact With IQGAP3 in Pan-Cancer

We employed the STRING database (https://string-db.org/cgi) to construct the protein interaction networks of IQGAP3 in cancer (Szklarczyk et al., 2017), and the GeneMANIA (http://genemania.org/) was employed to analyze the interaction gene with the IQGAP3 (Franz et al., 2018).

### Analysis of the Immunological Roles of IQGAP3 in Pan-Cancer

We employed the TIMER (https://cistrome.shinyapps.io/timer/) and XCELL tools (https://xcell.ucsf.edu/) to analyze the immunological roles of IQGAP3 (Li et al., 2017; Aran et al., 2017), including the correlation between diverse immune cells and the immune regulator. The TISIDB (http://cis.hku.hk/TISIDB/) was adopted to analyze the relationship between IQGAP3 expression and 28 tumor-infiltrating lymphocytes, 45 immune stimulators, 24 immune inhibitors, 41 chemokines, 18 receptors, and 21 MHC molecules in pan-cancer (Ru et al., 2019). The TMB and MSI scores were obtained from TCGA. Correlation analysis between the IQGAP3 expression and TMB or MSI was performed using Spearman’s method.

### Analysis of the Correlation Between IQGAP3 Expression and Drug Sensitivity

We employed the Genomics of Drug Sensitivity in Cancer (GDSC) (www.cancerRxgene.org) and CTRP databases to analyze the correlation between IQGAP3 expression and drug sensitivity (Basu et al., 2013; Yang et al., 2013).

### Analysis of the Molecular Characteristics of IQGAP3

We employed the lncLocator (www.csbio.sjtu.edu.cn/bioinf/lncLocator) and CPC2 (http://cpc2.cbi.pku.edu.cn) to examine the subcellular localization and the protein-coding ability of IQGAP3 (Kang et al., 2017; Cao et al., 2018).

### Cells and Cell Culture Conditions

The BEAS-2B cell line was purchased from the cell bank of Kunming Institute of Zoology and cultured in BEGM media (Lonza, CC-3170). HEK-293T was obtained from ATCC. Lung cancer cell lines, including A549, H1299, and H1975 were purchased from Cobioer, China, with the STR document, and the A549, H1299, and H1975 cells were all cultured in an RPMI1640 medium (Corning) supplemented with 10% fetal bovine serum (Cat# 10099141C, Gibco, United States) and 1% penicillin/streptomycin.

### Quantitative Real-Time PCR

The qRT-PCR assay was performed as documented (Yang et al., 2013). The primer sequences are as follows: IQGAP3-F: GCA​GCC​TAT​GAA​CGC​CTC​A, IQGAP3-R: GGA​GGG​TGC​AAA​ACA​GTG​G, β-actin-F: CTTCGCGGGCGACGAT, and β-actin-R: CCA​TAG​GAA​TCC​TTC​TGA​CC. The expression quantification was obtained with the 2−ΔΔCt method.

### Immunohistochemistry Staining

Immunohistochemistry staining assay was performed as documented (Basu et al., 2013). Briefly, lung cancer tissue and normal lung tissues were obtained from advanced-stage lung cancer patients from The Third Affiliated Hospital of Kunming Medical University (Yunnan Tumor Hospital), Kunming, China. These tissues were used to perform immunohistochemistry (IHC), primary antibody overnight incubation, and second antibody incubation, and finally, developed using the instrument. The detailed information of antibodies employed in our study is as follows: IQGAP3 antibody (Rabbit polyclonal to IQGAP3, ab219354, 1:500).

### Statistical Analysis

Analysis of the IQGAP3 expression pan-cancer was estimated using t-tests. For survival analysis, the HR and *p*-value were calculated employing univariate Cox regression analysis. Kaplan–Meier analysis was employed to examine the survival time of patients stratified according to high or low levels of the IQGAP3 expression. *p*-values less than 0.05 were considered statistically significant. For all figures, ∗, ∗∗, and ∗∗∗ indicate *p* < 0.05, *p* < 0.01, and *p* < 0.001, respectively.

## Results

### IQGAP3 has High Expression in Human Cancer

**FIGURE 1:**
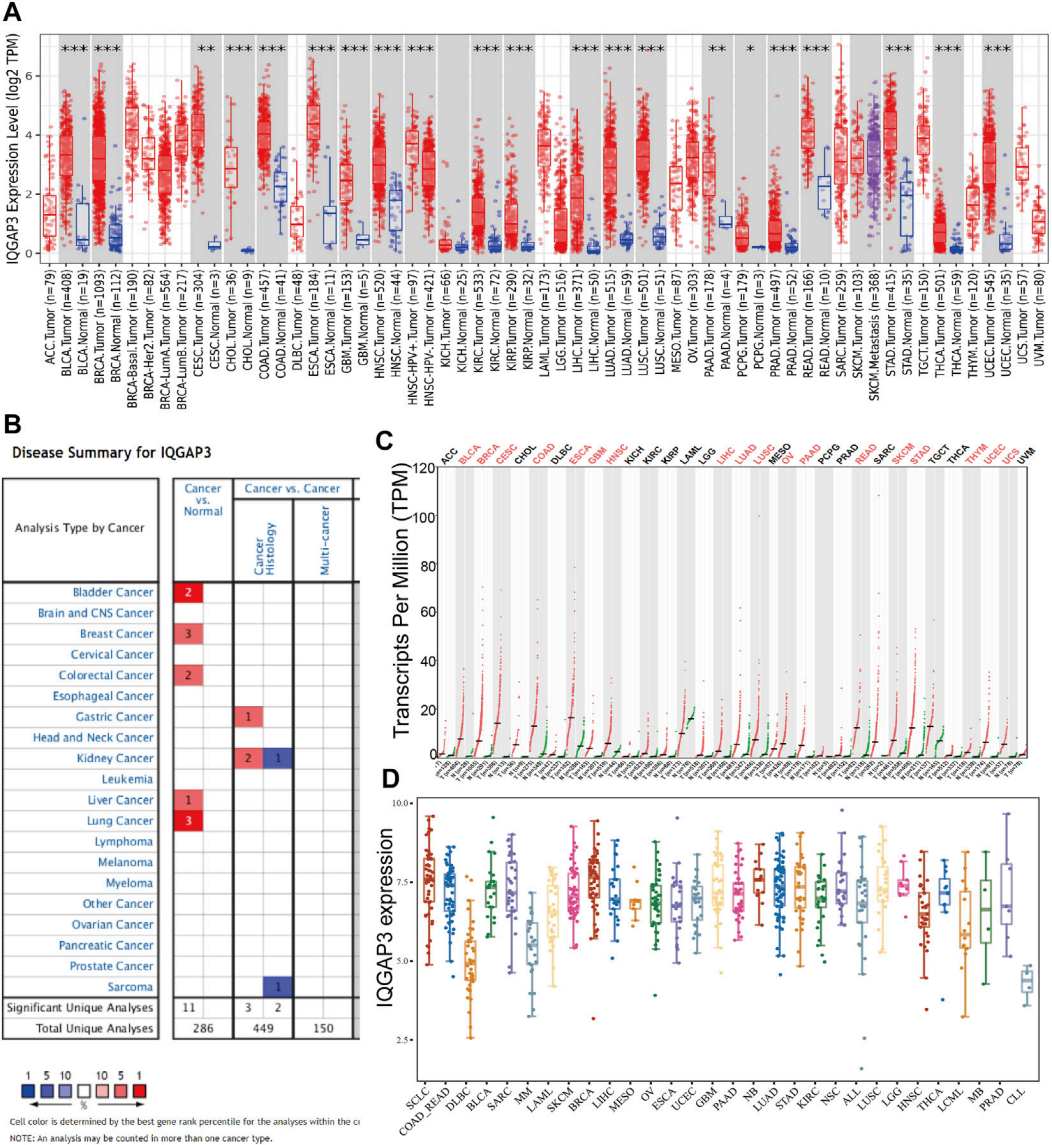
Expression analysis for IQGAP3 in human cancers. **(A)** The expression of IQGAP3 in pan-cancer analysis by the TIMER database. **(B)** The expression of IQGAP3 in pan-cancer analysis by using the oncomine database. **(C)** The expression of IQGAP3 in pan-cancer analysis by using the GEPIA database. **(D)** The expression of IQGAP3 in pan-cancer cells lines analysis by using the CCLE database.

To examine IQGAP3 expression in different types of human cancer, we first employed the TIMER database (http://timer.comp-genomics.org/). We discovered that IQGAP3 had high expression in BLCA, BRCA, CESC, CHOL, COAD, ESCA, GBM, HNSC, KIRC, KIRP, LUAD, LUSC, PAAD, PCPG, PRAD, READ, STAD, THCA, and UCEC (Figure 1A). We also analyzed IQGAP3 expression in human cancer based on Gene Expression Omnibus (GEO) datasets by employing the Oncomine database (www.oncomine.com/). IQGAP3 expression was increased in the cancer of the bladder, breast, colorectum, stomach, kidney, liver, and lung (Figure 1B). Owing to a lack of data for normal tissue in The Cancer Genome Atlas (TCGA) database (www.cancer.gov/about-nci/organization/ccg/research/structural-genomics/tcga/), we used the GEPIA database (http://gepia.cancer-pku.cn/) to explore IQGAP3 expression in human cancer types. A high expression of IQGAP3 was observed in BLCA, BRCA, CESC, COAD, ESCA, GBM, HNSC, LIHC, LUAD, LUSC, OV, PAAD, READ, SKCM, STAD, Thymoma (THYM), UCEC, and uterine carcinosarcoma (UCS) (Figure 1C). Finally, to determine IQGAP3 expression in different cancer cells lines, we used the Cancer Cell Line Encyclopedia (CCLE) database (https://sites.broadinstitute.org/ccle/). We found that IQGAP3 expression was upregulated in different cancer cells lines (Figure 1D). Overall, these results showed that IQGAP3 expression was upregulated in different types of human cancer.

### Correlation Between IQGAP3 Expression and the Pathological Stage

**FIGURE 2:**
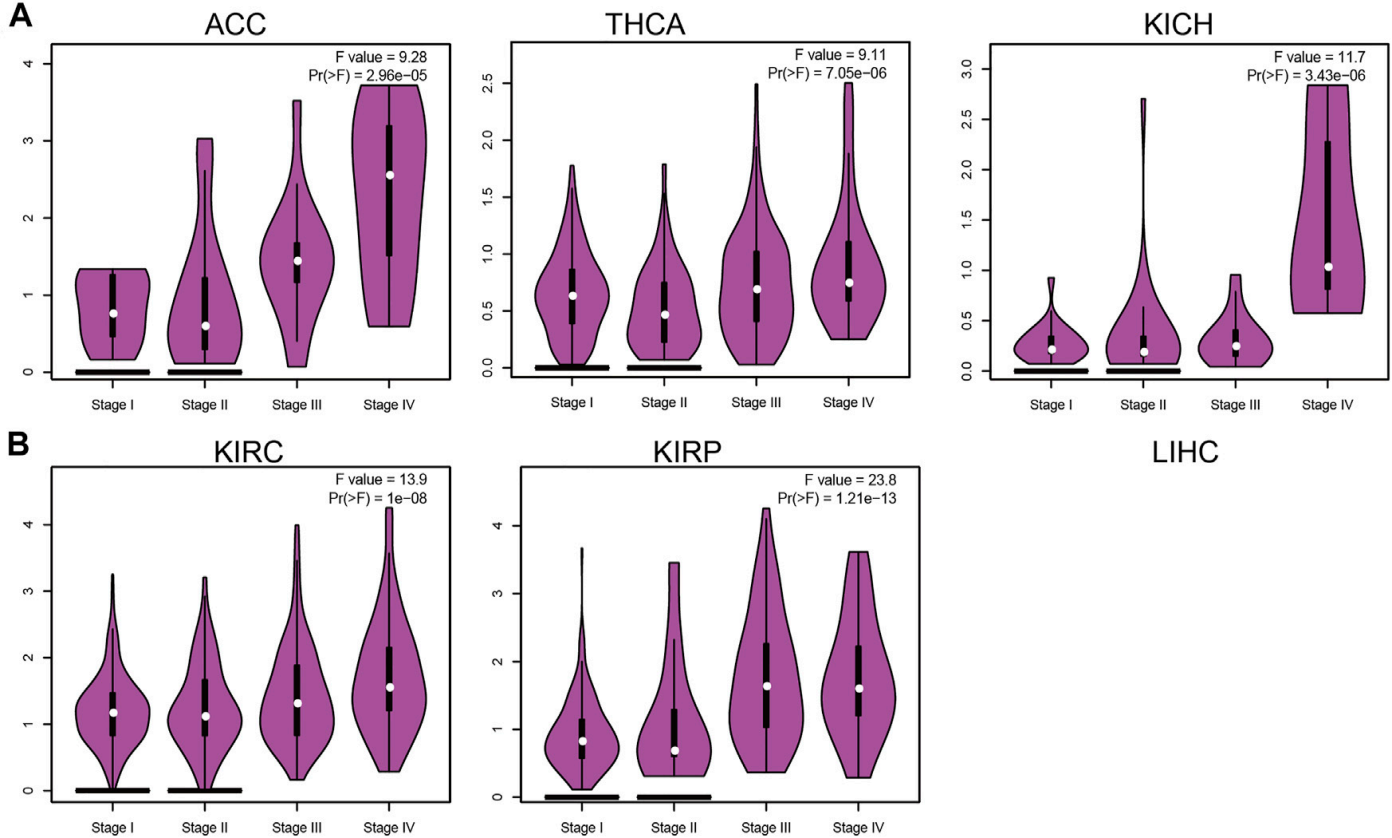
Analysis of the tumor stage for IQGAP3 in human cancers. **(A)** Analysis of the tumor stage for IQGAP3 in adrenocortical carcinoma, thyroid carcinoma, and kidney chromophobe, and **(B)** analysis of the tumor stage for IQGAP3 kidney renal clear cell carcinoma, and kidney renal papillary cell carcinoma by using the GEPIA database.

IQGAP3 expression was associated significantly with the pathological stage of ACC, BRCA, KICH, KIRC, KIRP, LIHC, LUAD, LUSC, OV, PAAD, and THCA (Figure 2 and Supplementary Figure S1). These findings demonstrated that IQGAP3 expression was correlated significantly with the pathological stage of different types of cancer.

### Correlation Between IQGAP3 Expression and Lymph Node Metastasis

Lymph node metastasis plays a crucial part in cancer progression. We examined IQGAP3 expression in lymph node metastasis of different types of cancer. We discovered that IQGAP3 expression was positively correlated with lymph node metastasis of BLCA, BRCA, CESC, CHOL, COAD, ESCA, HNSC, KICH, KIRC, KIRP, LIHC, LUAD, LUSC, MESC, PAAD, PRAD, READ, STAD, and THCA (Supplementary Figures S2A–C). These findings showed that IQGAP3 expression was significantly related to lymph node metastasis in different types of cancer.

### Prognostic Role of IQGAP3 in Human Cancers

**FIGURE 3:**
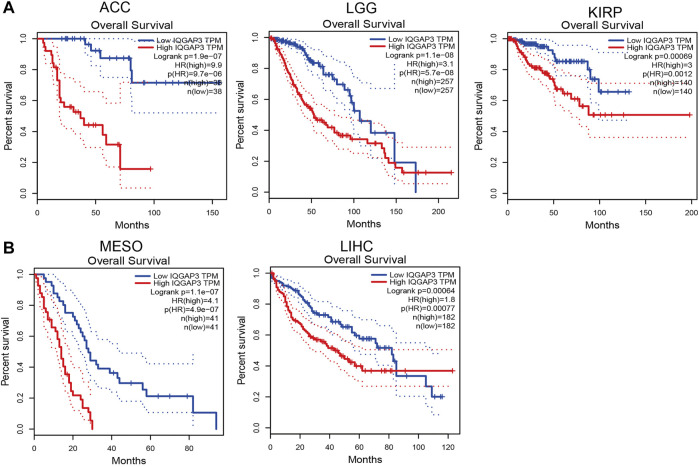
Analysis of the Overall survival for IQGAP3 in human cancers. **(A)** The overall survival for IQGAP3 in adrenocortical carcinoma, brain lower grade glioma, and kidney renal clear cell carcinoma analysis by using the GEPIA database, and **(B)** the overall survival for IQGAP3 in mesothelioma and liver hepatocellular carcinoma analysis by using the GEPIA database.

**FIGURE 4:**
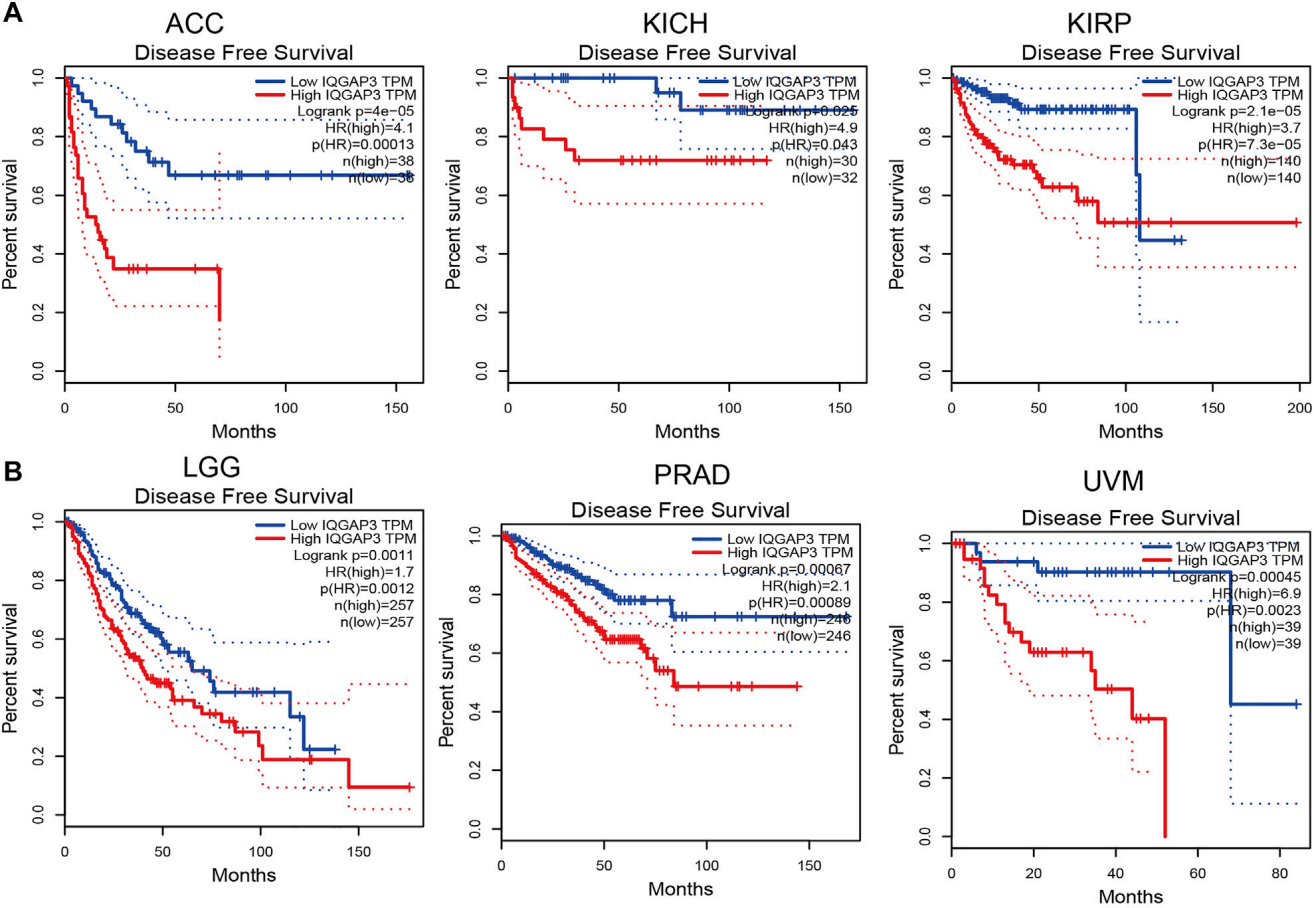
Analysis of the disease-free survival for IQGAP3 in human cancers. **(A)** The disease-free survival for IQGAP3 in adrenocortical carcinoma, kidney chromophobe, and kidney renal papillary cell carcinoma analysis by using the GEPIA database, and **(B)** the disease-free survival for IQGAP3 in brain lower grade glioma, prostate adenocarcinoma, and uveal melanoma analysis by using the GEPIA database.

To ascertain the prognostic role of IQGAP3 in different types of cancer, we ascertained the OS, DFS, progression-free survival (PFS), disease-specific survival (DSS), and relapse-free survival (RFS) in human cancer types. A high expression of IQGAP3 was not only related to poor OS in ACC, KIRC, KIRP, LAML, LGG, LIHC, LUAD, MESO, and UVM (Figure 3 and Supplementary Figure S3) but also associated with poor DFS in ACC, KICH, KIRP, LGG, LIHC, MESO, PRAD, SKCM, and UVM (Figure 4). Cox regression analysis showed that a high expression of IQGAP3 was related to poor PFS in ACC, KICH, KIRC, KIRP, LGG, LIHC, MESO, PAAD, PCPG, PRAD, THCA, UCEC, and UVM (Supplementary Figure S4). A high expression of IQGAP3 was associated with poor DSS in KIRP, LIHC, PAAD, PRAD, THCA, and UCEC (Supplementary Figure S4). To verify the results shown above, we employed a prognostic database (http://dna00.bio.kyutech.ac.jp/PrognoScan/index.html) to analyze the OS, RFS, and DFS in diverse GEO cohorts: identical results were obtained (Table 1). Collectively, these data indicated that IQGAP3 expression was closely related to the prognosis of patients with different cancer types.

**TABLE 1:** The prognosis of IQGAP3 in pan-cancer analysis by using the prognostic database.

Dataset	Cancer type	Endpoint	COX *p*-Value
GSE13507	Bladder cancer	DSS	0.00011324
GSE17536	Colorectal cancer	OS	0.000821988
GSE13507	Bladder cancer	OS	0.0016971
GSE4412	Brain cancer	OS	0.0027539
GSE17536	Colorectal cancer	DSS	0.00355702
GSE17537	Colorectal cancer	DFS	0.00799641
GSE8894	Lung cancer	RFS	0.00934389
GSE22138	Eye cancer	DFS	0.0150124
GSE3141	Lung cancer	OS	0.0154456
GSE31210	Lung cancer	RFS	0.0216044
GSE9891	Ovarian cancer	OS	0.0345845
GSE17536	Colorectal cancer	DFS	0.0357503
GSE31210	Lung cancer	OS	0.0361929
GSE17537	Colorectal cancer	DSS	0.0381264
GSE13213	Lung cancer	OS	0.0407668
GSE14333	Colorectal cancer	DFS	0.0453652
GSE1456-	Breast cancer	DSS	0.0525242

### IQGAP3 May Act as a Potential Biomarker in Human Cancers

We showed above that a high expression of IQGAP3 was correlated with the prognosis of patients with different cancer types. Next, we investigated if IQGAP3 could act as a biomarker for different cancer types. We undertook an analysis of receiver operating characteristic (ROC) curves of IQGAP3 expression to obtain the area under the ROC curve (AUC) values. The AUC values for BRCA, PRAD, LUSC, LUAD, KIRP, KIRC, COAD, READ, THCA, GBM, LGG, PAAD, SKCM, LIHC, STAD, and ESCA are given in Supplementary Figure S5. The latter showed that IQGAP3 could be used as a biomarker to diagnose different types of cancer with high sensitivity and specificity.

### Relationship Between IQGAP3 Expression and Immune Subtypes and Molecular Subtypes in Human Cancers

According to different features, human cancers can be divided into immune subtypes and molecular subtypes. Immune subtypes can be classified further into six types: C1 (wound healing), C2 (interferon-gamma dominant), C3 (inflammatory), C4 (lymphocyte depleted), C5 (immunologically quiet), and C6 (transforming growth factor-β dominant) (Hu et al., 2021). IQGAP3 showed high expression in C1 and C2 and low expression in C3 in LUAD (Supplementary Figure S6). For molecular subtypes, IQGAP3 displayed different expressions in different cancer types (Supplementary Figure S7). These results suggested that IQGAP3 had different expression patterns in human cancer types.

### Correlation Between IQGAP3 Expression and TMB and MSI

**FIGURE 5:**
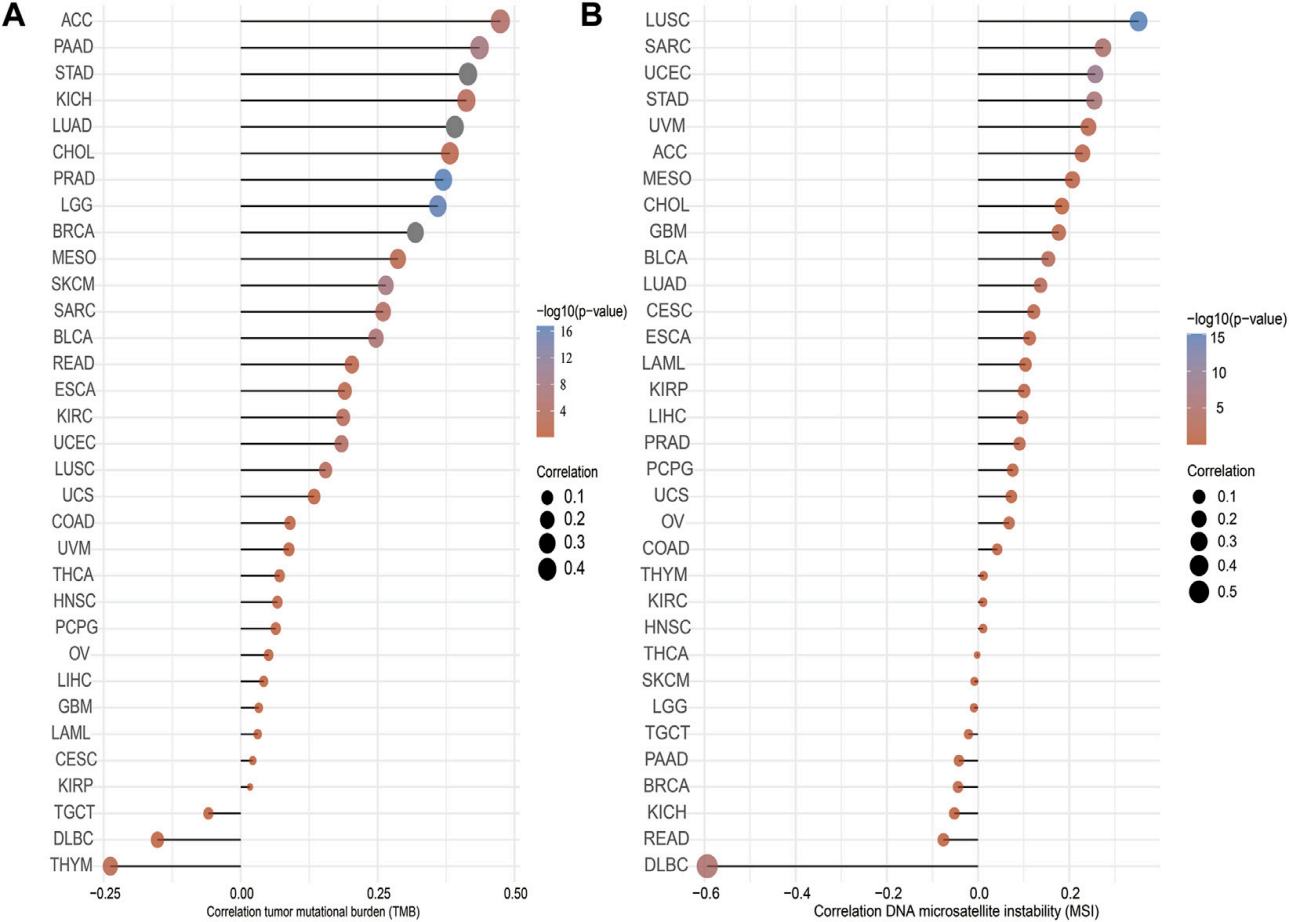
Analysis of the correlation between the IQGAP3 and TMS, MSI. **(A)** Analysis of the correlation between IQGAP3 and TMS. **(B)** Analysis of the correlation between IQGAP3 and MSI.

TMB is the number of noninherited mutations per million bases of an investigated genomic sequence. TMB has emerged as a specific and sensitive biomarker of the response to immune checkpoint inhibitors (Addeo et al., 2021). We examined the correlation between IQGAP3 expression and TMB of human cancers. IQGAP3 expression was markedly positively correlated with TMB of ACC, PAAD, STAD, KICH, LUAD, CHOL, and PRAD and negatively correlated with TMB of DLBC and THYM (Figure 5A).

MSI represents a hyper-mutable state of DNA sequences caused by a lack of activity of DNA repair (Boland and Goel, 2010). We explored the correlation between IQGAP3 expression and MSI in human cancers. IQGAP3 expression was markedly positively correlated with MSI in LUSC, SRAC, and UCEC and negatively correlated with MSI in DLBC (Figure 5B). Collectively, these data implied that IQGAP3 may influence antitumor immunity by regulating the composition and immune mechanism in the tumor microenvironment.

### Genetic Alteration of IQGAP3 in Human Cancers

**FIGURE 6:**
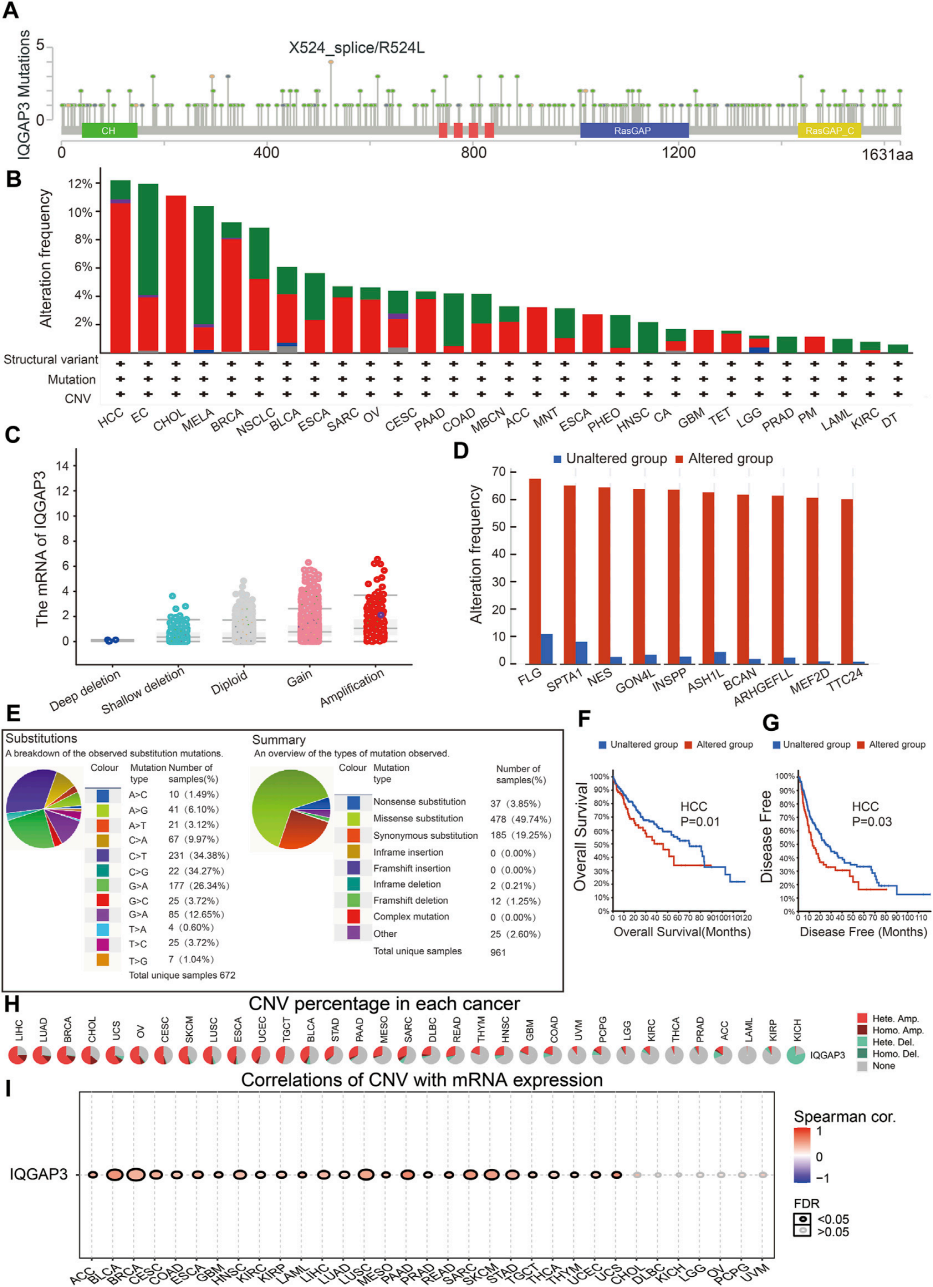
Analysis of the gene mutation of IQGAP3 in pan-cancer. **(A)** Representation of IQGAP3 mutations (TCGA) in diverse human cancers by using the cBioPortal database. **(B)** The mutation frequency of IQGAP3 in pan-cancer was examined by employing the cBioPortal database; red: amplification, green: mutation, blue: deep deletion, and purple: structural variant. **(C)** The correlation between the gene mutation of IQGAP3 and its expression in pan-cancer was examined by employing the cBioPortal database. **(D)** The correlation between the gene mutation of IQGAP3 and the closest gene in pan-cancer was examined by employing the CIO portal database. **(E)** The percentages of mutation types of IQGAP3 in pan-cancer were indicated in a pie chart examined by employing the Catalogue of Somatic Mutations. **(F)** The mutation of IQGAP3 affects the OS of HCC patients examined by employing the cBioPortal database. **(G)** The mutation of IQGAP3 affects the DFS of HCC patients examined by employing the cBioPortal database. **(H)** The CNV of IQGAP3 in diverse human cancer by using the GSCA database. **(I)** The correlation between the CNV of IQGAP3 and its expression in pan-cancer was examined by using the GSCA database.

We wished to explore the gene-mutation information of IQGAP3 in human cancer types. We used the cBioPortal (www.cbioportal.org/) database, and the main information is shown in Figure 6A. The frequency of IQGAP3 alterations (>12%) was the highest in hepatocellular carcinoma, with amplification being the main type of alteration (Figure 6B). Amplification was the main reason why the mRNA of IQGAP3 was upregulated in different cancer types (Figure 6C). IQGAP3 mutation was significantly correlated with genes such as FLG, SPTA1, NES, GON4L, INSPP, ASH1L, BCAN, ARHGEFLL, MEF2D, and TTC24 (Figure 6D).

We also examined the mutation type and base mutation in cancer types. A missense substitution and base mutation C > T were the most common in different cancer types (Figure 6E). The mutation of IQGAP3 affected the prognosis of patients with hepatocellular carcinoma, with a poor OS and DFS being noted (Figures 6F, G). Finally, we showed that the CNV of IQGAP3 in different cancer types was positively correlated with its expression (Figures 6H, I and Supplementary Table S1). Overall, these results suggested that the CNV of IQGAP3 was positively correlated with IQGAP3 expression in different types of human cancer.

### Level of DNA Methylation and IQGAP3 Expression in Human Cancers

DNA methylation has a significant role in the regulation of gene expression (Klutstein et al., 2016). Next, we explored if a high expression of IQGAP3 was attributed to low DNA methylation in the promoter region of IQGAP3. We discovered that DNA hypo-methylation in IQGAP3 was negatively correlated with IQGAP3 expression in ACC, BLCA, BRCA, CHOL, COAD, KIRC, LGG, LIHC, LUAD, LUSC, PAAD, READ, SARC, SKCM, STAD, testicular germ cell tumors (TGCT), THCA, UCEC, UCS, and UVM (Supplementary Table S2). We also examined if the methylation level of IQGAP3 affected the prognosis of patients with different types of cancer. A low DNA-methylation level in IQGAP3 was correlated with a better poor prognosis in different cancer types (Table 2). These findings demonstrated that IQGAP3 expression was significantly correlated with DNA methylation in different types of human cancer.

**TABLE 2:** Analysis of the correlation between the DNA methylation of IQGAP3 and prognosis of cancer patients.

DNA methylation sites	Cancer	HR	CI	*p-*Value
cg12617080	LGG	0.327	(0.228; 0.469)	1.22E-09
cg23679769	SKCM	0.498	(0.375; 0.663)	1.72E-06
cg26024851	SKCM	0.47	(0.331; 0.668)	2.41E-05
cg12262564	SKCM	0.476	(0.334; 0.678)	3.94E-05
cg12124478	SKCM	0.504	(0.36; 0.706)	6.74E-05
cg12617080	ACC	4.808	(2.089; 11.07)	0.000223
cg12262564	CESC	0.426	(0.262; 0.693)	0.000577
cg26024851	UVM	4.626	(1.93; 11.091)	0.000597
cg12124478	BRCA	0.524	(0.35; 0.782)	0.001591
cg12617080	KIRC	0.528	(0.35; 0.796)	0.002304
cg12689752	SKCM	0.627	(0.461; 0.851)	0.002774
cg12689752	LGG	0.577	(0.402; 0.829)	0.002958
cg12617080	UVM	0.262	(0.108; 0.636)	0.003081
cg12124478	CESC	0.465	(0.277; 0.782)	0.003912
cg26024851	LGG	1.722	(1.189; 2.494)	0.004025
cg12262564	UVM	3.398	(1.435; 8.046)	0.005408
cg26024851	READ	0.253	(0.093; 0.688)	0.007106
cg12441221	LIHC	0.596	(0.407; 0.872)	0.007712
cg12262564	BRCA	0.484	(0.284; 0.827)	0.00789
cg12262564	LGG	1.624	(1.128; 2.337)	0.009069
cg12689752	GBM	0.581	(0.385; 0.878)	0.009859
cg12617080	LAML	1.614	(1.12; 2.328)	0.010271
cg12441221	SKCM	0.687	(0.515; 0.918)	0.01104
cg26024851	PAAD	1.825	(1.128; 2.951)	0.014228
cg12441221	ACC	0.382	(0.174; 0.841)	0.016835
cg12617080	GBM	0.58	(0.366; 0.92)	0.020547
cg23679769	HNSC	0.731	(0.559; 0.956)	0.02211
cg12441221	LAML	0.577	(0.359; 0.926)	0.02262
cg23679769	BRCA	0.56	(0.34; 0.922)	0.022725
cg12617080	PAAD	0.566	(0.345; 0.928)	0.02403
cg12262564	HNSC	0.717	(0.536; 0.959)	0.025059
cg26024851	HNSC	0.689	(0.495; 0.959)	0.027125
cg17722719	LIHC	0.648	(0.44; 0.953)	0.027507
cg26024851	GBM	0.608	(0.385; 0.96)	0.032803
cg12262564	KIRC	0.581	(0.35; 0.966)	0.036367
cg26024851	CESC	0.584	(0.351; 0.972)	0.038513
cg17722719	KIRP	0.513	(0.273; 0.966)	0.038718
cg12124478	SARC	0.656	(0.439; 0.98)	0.039613
cg12617080	STAD	0.65	(0.431; 0.981)	0.040199
cg17722719	LAML	0.676	(0.464; 0.985)	0.041583
cg12124478	UVM	2.369	(1.033; 5.433)	0.041747
cg17722719	ACC	0.338	(0.117; 0.975)	0.044811
cg12262564	BLCA	0.734	(0.541; 0.995)	0.046522
cg12617080	LIHC	0.681	(0.467; 0.994)	0.046564
cg12441221	LUAD	1.375	(1.004; 1.884)	0.047151
cg23679769	KIRC	0.606	(0.368; 0.997)	0.048554
cg12689752	BLCA	1.372	(1; 1.882)	0.049922

### IQGAP3 Functions in Human Cancers

**FIGURE 7:**
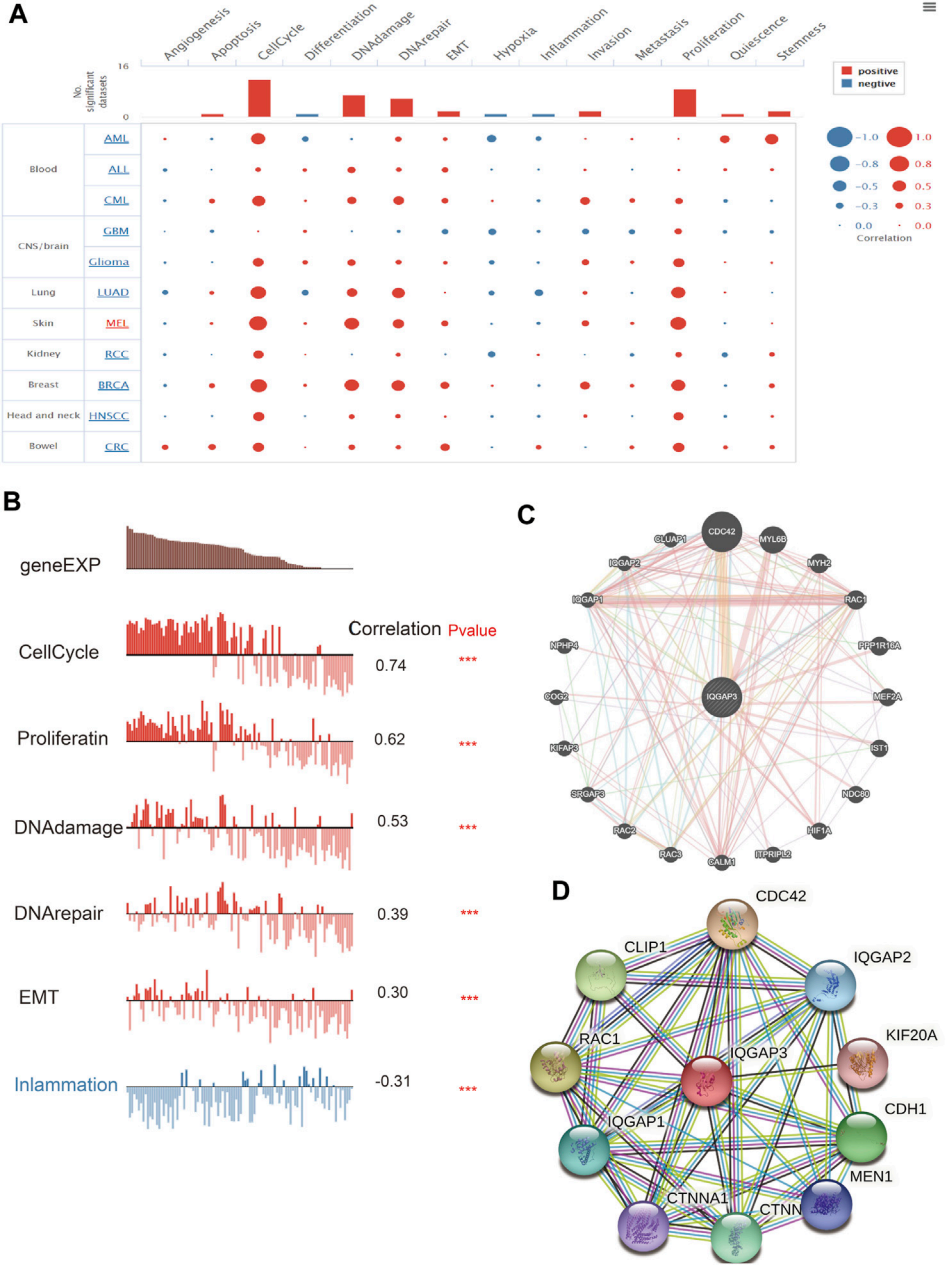
Analysis of the function for IQGAP3 in human cancers. **(A)** The function of IQGAP3 in pan-cancer analysis by using the CancerSEA database. **(B)** The correlation between the IQGAP3 and diverse function analysis by using the CancerSEA database. **(C)** The gene interaction meshwork of IQGAP3 was constructed using GeneMania. **(D)** The STRING database was employed to construct the protein interaction meshwork of IQGAP3.

After discovering that IQGAP3 was correlated markedly to the prognosis, tumor stage, and lymph node metastasis, we explored IQGAP3 functions in human cancer types using CancerSEA (http://biocc.hrbmu.edu.cn/). IQGAP3 was involved mainly in angiogenesis, apoptosis, cell cycle, cell differentiation, DNA damage, epithelial–mesenchymal transition (EMT), hypoxia, inflammation, invasion, metastasis, and proliferation in human cancers (Figure 7A). A high expression of IQGAP3 was positively correlated with the cell cycle (r = 0.74), cell proliferation (r = 0.62), DNA damage (r = 0.53), DNA repair (r = 0.39), EMT (r = 0.30) and inflammation (r = –0.30) in different cancer types (Figure 7B). These findings suggested that IQGAP3 has a pivotal role in the initiation and prognosis of diverse types of human cancer.

We employed the GeneMANIA (https://genemania.org/) and Search Tool for the Retrieval of Interacting Genes/Proteins (STRING; https://string-db.org/) databases to construct gene-interaction and protein-interaction networks with IQGAP3. The genes most closely associated with IQGAP3 were those of CDC42, MYL6B, MYH2, RAC1, PPP1R16A, MEF2A, IST1, NDC80, HIF1A, ITPRIPL2, CALM1, RAC3, RAC2, SRGAP3, KIFAP3, COG2, NPHP4, IQGAP1, IQGAP2, and CLUAP1 (Figure 7C). The proteins most closely associated with IQGAP3 were those for CDC42, IQGAP2, KIF20, CDH1, MEN1, CTNNB1, CTNNA1, IQGAP1, RAC1, and CLIP1 (Figure 7D). These proteins have been reported (Modhukur et al., 2018) to have crucial roles in the cell proliferation and cell cycles of different cancer types.

### CNV and DNA Methylation of Proteins That Interact With IQGAP3 in Human Cancer

We wished to examine the expression pattern of genes that interact with IQGAP3. We employed the Gene Set Cancer Analysis (GSCA) (http://bioinfo.life.hust.edu.cn/GSCA/#/) database to analyze the CNV and DNA methylation of genes that interact with IQGAP3 in different cancer types.

**FIGURE 8:**
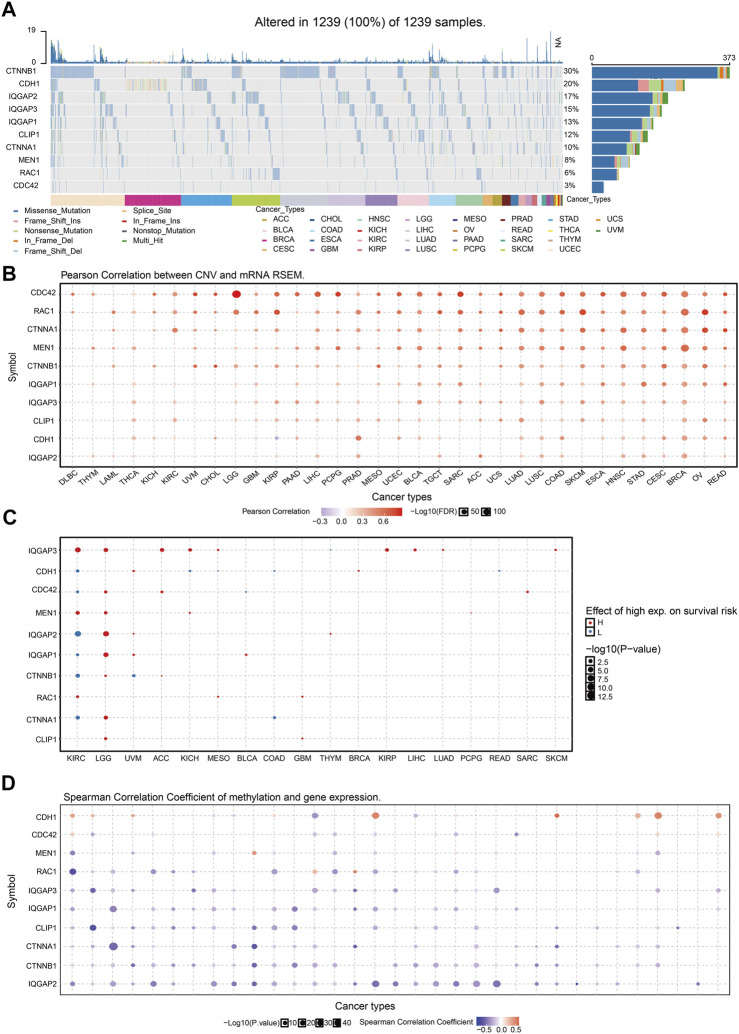
Analysis of the mutation for IQGAP3 interaction with the gene in human cancers. **(A)** The mutation of IQGAP3 interaction with the gene in human cancers was analyzed by using the GSCA tools. **(B)** The correlation between CNV and expression of IQGAP3 interaction with the gene in human cancers was analyzed by using the GSCA tools. **(C)** The correlation between the prognosis and CNV of IQGAP3 interaction with the gene in human cancers was analyzed by using the GSCA tools. **(D)** The correlation between DNA methylation and expression of IQGAP3 interaction with the gene in human cancers was analyzed by using the GSCA tools.

*CTNNB1* had the highest mutation rate (29%) (Figure 8A), and the CNV was markedly positively correlated with *CTNNB1* expression in human cancers (Figure 8B). The gene CNV of IQGAP3 interaction genes significantly affected the prognosis of diverse patients (Figure 8C). Next, we examined the DNA-methylation level of genes in different cancer types. DNA methylation of these genes was significantly negatively correlated with mRNA expression in different cancer types (Figure 8D).

### IQGAP3 and Enrichment of Signaling Pathways in Human Cancer

We wished to explore if an “IQGAP3 axis” plays an important part in cancer progression. We used the LinkedOmics (www.linkedomics.org/) database and Kyoto Encyclopedia of Genes and Genomes (www.genome.jp/kegg/) database to analyze which signaling pathways were enriched in different cancer types.

**FIGURE 9:**
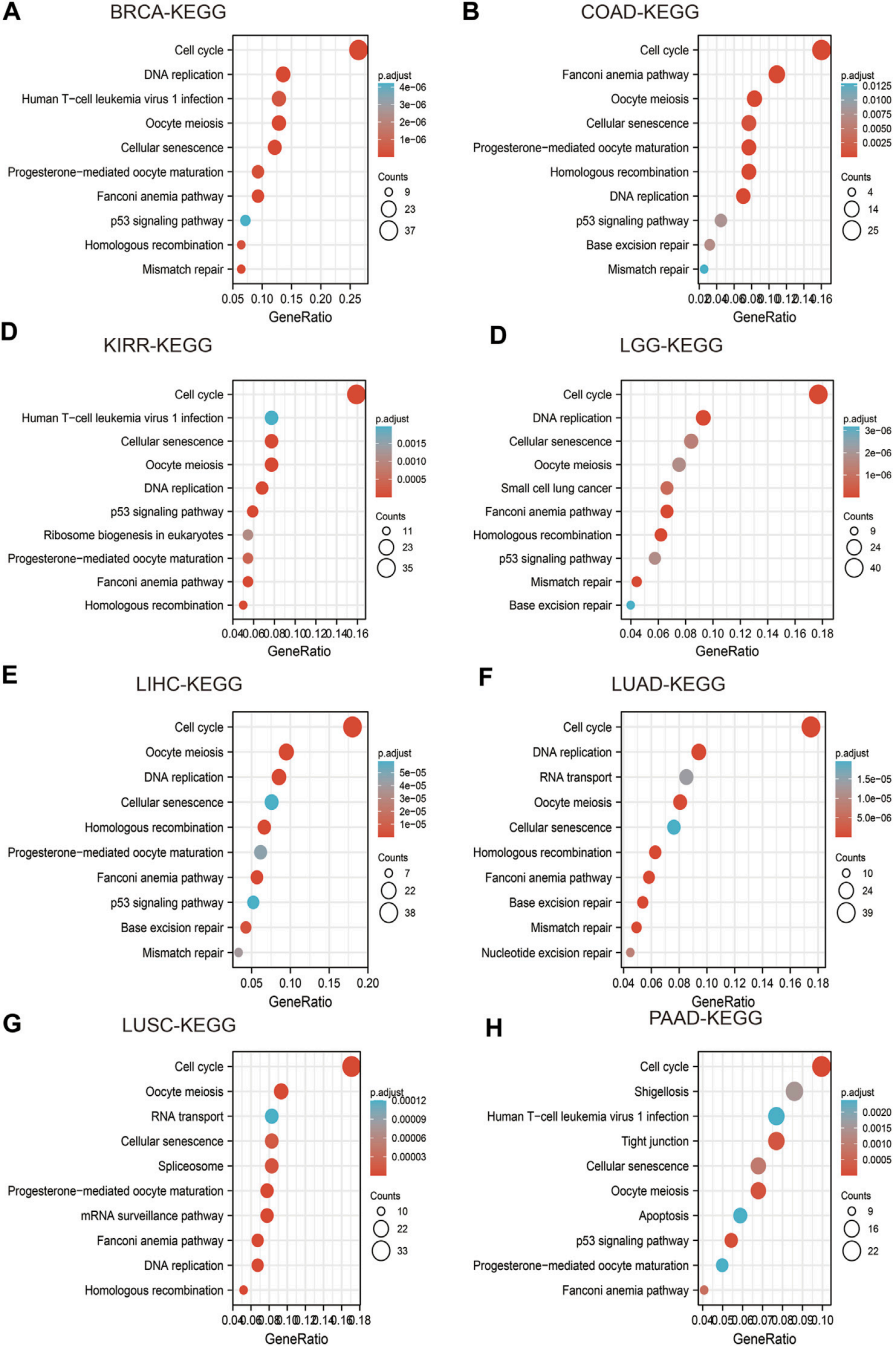
Analysis of the signaling pathway for IQGAP3 in human cancers. **(A–D)** The KEGG pathway of IQGAP3 in BRCA, COAD, KIRP, and LGG was analyzed by using LinkedOmics. **(E–H)** The KEGG pathway of IQGAP3 in LIHC, LUAD, LUSC, and PAAD analysis by LinkedOmics.

High expression of IQGAP3 was mainly involved in “cell cycle,” “ECM-receptor interaction,” and “miRNA in cancer” in BRCA (Figure 9A); “spliceosome” and “human T cell virus” in COAD (Figure 9B); “cell cycle” and “lysosomes” in KIRP (Figure 9C); “PI3K-AKT signaling pathway” and “rap1 signaling pathway” in LGG (Figure 9D); “cell cycle” and “RNA transport” in LIHC (Figure 9E) “cell cycle,” “spliceosome,” and “RNA transport” in LUAD (Figure 9F); “cell adhesion” and “cell cycle” in LUSC (Figure 9G); “Nod-like receptor signaling pathway” and “tight junctions” in PAAD (Figure 9H). These results demonstrated that IQGAP3 has a crucial role in the development of different cancer types.

### Transcription Factors of IQGAP3 in Human Cancers

Transcription factors have indispensable roles in controlling gene expression (Bradner et al., 2017). We explored the transcription factors that could regulate IQGAP3 transcription. We employed the JASPAR (https://jaspar.genereg.net/), PROMO (http://alggen.lsi.upc.es/cgi-bin/promo_v3/), ConTrav3 (http://bioit2.irc.ugent.be/contra/v3/#/step/1/), and UCSC (https://genome.ucsc.edu/) databases. PTBP1 was positively correlated with IQGAP3 expression in diverse cancer types (Supplementary Figures S8A–8D and Table 3). We used the KNOCK-TF (www.licpathway.net/KnockTF/) database to verify the above result. The depletion of PTBP1 reduced IQGAP3 expression in LIHC (Supplementary Figure S8E). A high expression of PTBP1 was correlated with a poor prognosis in patients with ESCA, KIRP, LIHC, LUAD, PAAD, or SARC, and related to better prognosis in patients with BLCA, ESCC, OV, READ, STAD, or THYM (Supplementary Figures S9A–9D). We examined GEO datasets and obtained identical results (Table 4). Overall, these results demonstrated that PTBP1 may be a transcription factor for IQGAP3 in different cancer types.

**TABLE 3:** The correlation between the PTBP1 and IQGAP3 expressions.

Cancer	Sample number	Coefficient-R	*p*-Value
STAD	375	0.677	1.06E-51
DLBC	48	0.61	4.24E-06
LGG	529	0.604	5.30E-54
HNSC	502	0.6	1.72E-50
READ	167	0.595	2.11E-17
THYM	119	0.583	3.46E-12
KICH	65	0.561	1.19E-06
LUAD	526	0.561	5.31E-45
LIHC	374	0.54	1.09E-29
UVM	80	0.528	4.96E-07
PAAD	178	0.522	7.64E-14
LUSC	501	0.51	1.38E-34
PRAD	499	0.485	9.54E-31
TGCT	156	0.485	1.34E-10
ACC	79	0.472	1.11E-05
BLCA	411	0.47	6.34E-24
MESO	86	0.442	2.04E-05
KIRP	289	0.429	2.29E-14
ESCA	162	0.428	1.30E-08
COAD	471	0.415	5.12E-21
PCPG	183	0.383	8.76E-08
UCEC	548	0.374	1.21E-19
OV	379	0.366	2.00E-13
BRCA	1,104	0.35	3.39E-33
SKCM	471	0.345	1.40E-14
LAML	151	0.337	2.31E-05
KIRC	535	0.327	8.59E-15
UCS	56	0.308	2.11E-02
CHOL	36	0.304	7.19E-02
SARC	263	0.303	5.55E-07
THCA	510	0.197	7.43E-06
CESC	306	0.187	1.02E-03

**TABLE 4:** The prognosis of IQGAP3 in pan-cancer analysis by using the prognostic database.

Dataset	Cancer type	Endpoint	COX *p*-Value
GSE31210	Lung cancer	RFS	1.26E-05
GSE31210	Lung cancer	RFS	0.0001
GSE30929	Soft tissue cancer	PDF	0.000186
GSE30929	Soft tissue cancer	DFS	0.000463
GSE4922	Breast cancer	PDF	0.000481
GSE30929	Soft tissue cancer	DFS	0.000582
GSE4271	Brain cancer	OS	0.000604
GSE2658	Blood cancer	DSS	0.00072
GSE30929	Soft tissue cancer	PDF	0.001089
GSE30929	Soft tissue cancer	DFS	0.001362
GSE31210	Lung cancer	RFS	0.002043
GSE13213	Lung cancer	OS	0.00284
GSE9891	Ovarian cancer	OS	0.002943
GSE7378	Breast cancer	DFS	0.003144
GSE4271	Brain cancer	OS	0.003199
GSE1456	Breast cancer	RFS	0.003842
GSE7378	Breast cancer	PDF	0.003893
GSE4271	Brain cancer	OS	0.00407
GSE4271	Brain cancer	OS	0.005597
GSE1456	Breast cancer	RFS	0.005869
GSE16581	Brain cancer	OS	0.005955
GSE9893	Breast cancer	OS	0.005994
GSE1456	Breast cancer	RFS	0.006665
GSE4922	Breast cancer	DFS	0.006734
GSE12276	Breast cancer	RFS	0.007262
GSE7378	Breast cancer	DFS	0.007317
GSE31210	Lung cancer	RFS	0.007478
GSE31210	Lung cancer	OS	0.00766
GSE13507	Bladder cancer	DSS	0.008043
GSE7378	Breast cancer	DFS	0.008527
GSE7378	Breast cancer	PDF	0.009135
GSE1456	Breast cancer	DSS	0.009382
GSE14764	Ovarian cancer	OS	0.009775
GSE1456	Breast cancer	DSS	0.010066
GSE1456	Breast cancer	RFS	0.010083
GSE3494	Breast cancer	DSS	0.010517
GSE1456	Breast cancer	DSS	0.01119
GSE31210	Lung cancer	RFS	0.012373
GSE1456	Breast cancer	OS	0.012975
GSE4271	Brain cancer	OS	0.013415
GSE3494	Breast cancer	DSS	0.01436
GSE31210	Lung cancer	OS	0.015052
GSE1456	Breast cancer	RFS	0.017259
GSE1456	Breast cancer	RFS	0.019606
GSE2658	Blood cancer	DSS	0.021756
GSE2658	Blood cancer	DSS	0.022514
GSE17537	Colorectal cancer	DSS	0.022708
GSE9195	Breast cancer	DFS	0.024236
GSE16131	Blood cancer	OS	0.027672
GSE1456	Breast cancer	DSS	0.029055
GSE19615	Breast cancer	DFS	0.029679
GSE2658	Blood cancer	DSS	0.031383
GSE16581	Brain cancer	OS	0.032206
GSE17537	Colorectal cancer	DFS	0.033958
GSE31210	Lung cancer	RFS	0.034197
GSE2658	Blood cancer	DSS	0.035098
GSE1456	Breast cancer	DSS	0.037992
GSE19234	Skin cancer	OS	0.039527
GSE11595	Esophagus cancer	OS	0.039893
GSE19234	Skin cancer	OS	0.040044
GSE1456	Breast cancer	DSS	0.040637
GSE17537	Colorectal cancer	OS	0.042439
GSE2990	Breast cancer	DFS	0.043585
GSE1456	Breast cancer	DSS	0.043756
GSE5287	Bladder cancer	OS	0.044084
GSE4922	Breast cancer	DFS	0.046047
GSE19234	Skin cancer	OS	0.046294
GSE31210	Lung cancer	OS	0.046761
GSE19234	Skin cancer	OS	0.047191
GSE19234	Skin cancer	OS	0.04904

### Upstream microRNAs of IQGAP3 in Human Cancers

miRs have crucial roles in regulating the expression of messenger (m)RNA. According to the competing endogenous RNA (ceRNA) hypothesis, there is a negative correlation between miR expression and mRNA expression. If IQGAP3 shows high expression in cancer, the miR expression in cancer should be low. We used public databases to analyze the upstream miRs of IQGAP3. Using different datasets to obtain intersections, we identified four miRs (miR-196b-5p, miR-422a, miR-18b-5p, let-7c-5p). Among these miRs, only the expression of let-7c-5p was decreased significantly in human cancers.

Next, we analyzed the expression of let-7c-5p in human cancers and investigated its prognostic value. Let-7c-5p had low expression in CHOL, BRCA, BLCA, UCEC, THCA, SATD, LUSC, LUAD, LIHC, KICH, HNSC, and COAD (Supplementary Figure S10 and Supplementary Table S3). A high expression of let-7c-5p was correlated with a good prognosis in patients with BRCA, CECS, ESCA, HNSC, KIRP, LIHC, LUAD, or LUSC, but correlated with a poor prognosis in patients with BLCA, PAAD, or STAD (Supplementary Figure S11). A low expression of let-7c-5p was correlated with the tumor stage in diverse cancer types (Supplementary Figure S12). These results showed that let-7c-5p expression was decreased in most cancer types and that increased expression of let-7c-5p was correlated with a better prognosis in many cancer types. We also analyzed the correlation between let-7c-5p and IQGAP3 in human cancers. Let-7c-5p expression was negatively correlated with IQGAP3 expression in human cancers (Supplementary Table S4). These results suggested that let-7c-5p was the most potential binding IQGAP3 in cancer patients.

### Upstream lncRNAs of Let-7c-5p in Human Cancers

**FIGURE 10:**
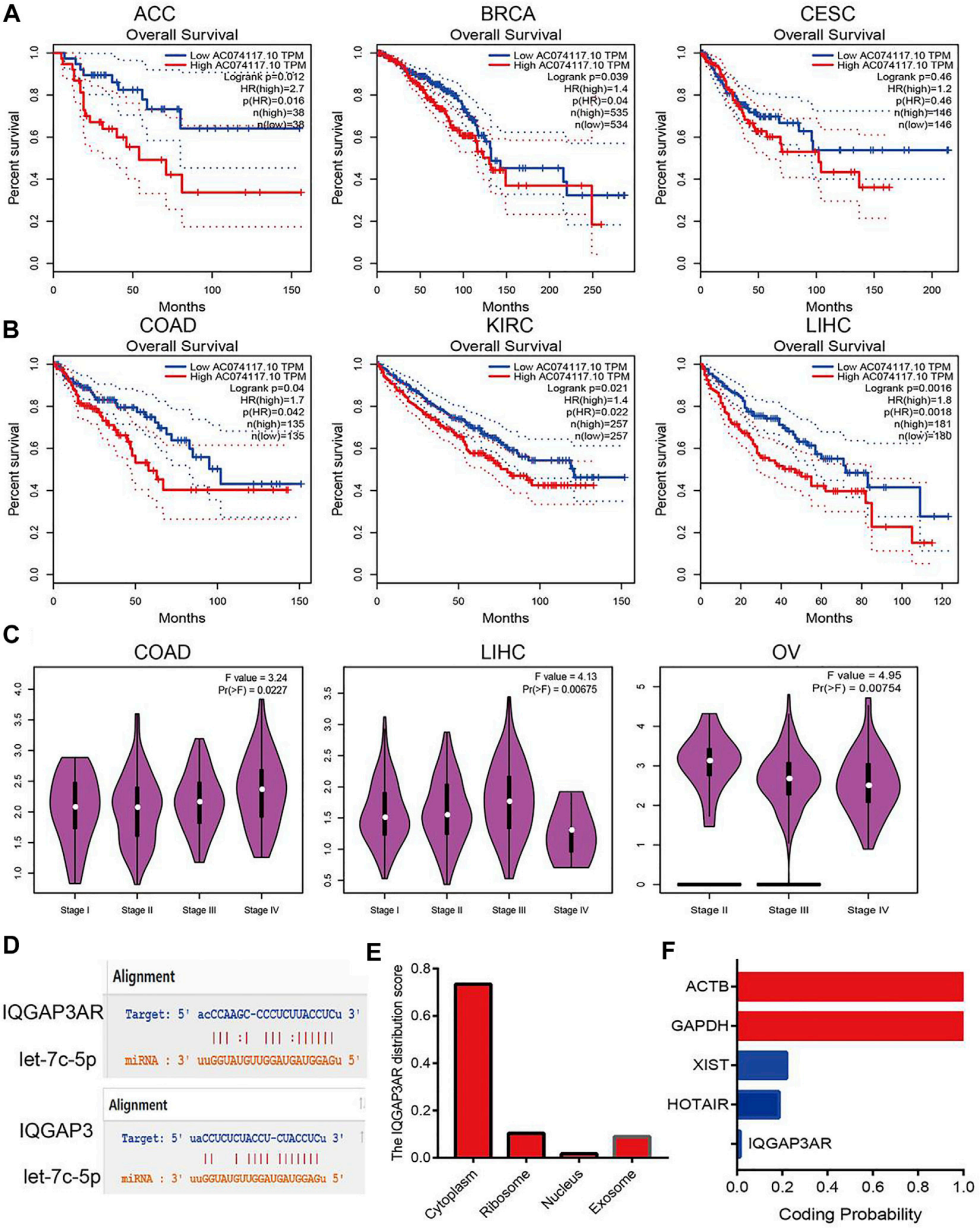
Analysis upstream lncRNA of let-7c-5p in pan-cancer. **(A)** The prognosis of IQGAP3AR in ACC, BRCA, and CESC was analyzed by using starBase. **(B)** The prognosis of IQGAP3AR in COAD, KIRC, and LIHC was analyzed by using starBase. **(C)** The correlation between the IQGAP3AR and tumor stage in COAD, LIHC, and OV was analyzed by using starBase. **(D)** The target sites between the IQGAP3, let-7c-5p, and IQGAP3 were predicted by using starBase. **(E)** The subcellular localization of IQGAP3 was analyzed by using the lncLocator tools. **(F)** The coding potential of IQGAP3 was analyzed by using the coding potential calculator.

We wished to identify the upstream lncRNAs of let-7c-5p in human cancers. We employed the starBase (https://starbase.sysu.edu.cn/) and LncRNAbase databases (http://carolina.imis.athena-innovation.gr/diana_tools/web/index.php?r=lncbasev2%2Findex-predicted) to predict the lncRNAs that may bind with let-7c-5p. We discovered five possible lncRNAs: AC234582.1, AL590666.2, AL590666.2, MIR29B2CHG, and IQGAP3AR. Among these lncRNAs, only the expression of IQGAP3AR was upregulated significantly in BRCA, BLCA, UCEC, STAD, PRAD, LUSC, LUAD, LIHC, KIRP, CHOL, KICH, HNSC, ESCA, and COAD (Supplementary Figures S13A–13D and Supplementary Table S5). We also identified a drug that was negatively correlated with IQGAP3AR expression (Supplementary Figure S13E). Further study revealed that the high expression of IQGAP3AR was not only associated with a poor prognosis in patients with ACC, BRCA, CESC, COAD, KIRC, or LIHC but also correlated with the tumor stage in COAD, LIHC, and OV. The expression of the other lncRNAs was not significantly different in different cancer types (Figures 10A–C).

According to the ceRNA hypothesis, lncRNA expression should have a negative correlation with let-7c-5p expression and positive correlation with IQGAP3 expression. The Spearman correlation analysis revealed IQGAP3AR expression to be significantly negatively correlated with let-7c-5p expression (Supplementary Figures S14A–14C and Supplementary Table S6) and positively correlated with the expression of IQGAP3 in pan-cancer (Supplementary Figures S15A–15C and Supplementary Table S7). The target sites between IQGAP3AR, let-7c-5p, and IQGAP3 were predicted by using TarBase (https://carolina.imis.athena-innovation.gr/) (Figure 10D).

The subcellular localization of IQGAP3AR was identified using the lncLocator (www.csbio.sjtu.edu.cn/bioinf/lncLocator/) database. IQGAP3AR was located mainly in the cytoplasm (Figure 10E). We also analyzed the protein-coding potential of IQGAP3AR by performing the coding potential calculator: IQGAP3AR did not possess the protein-coding ability (Figure 10F). Our results suggested that IQGAP3AR may be upstream of the lncRNA let-7c-5p, which regulates IQGAP3 expression in different cancer types.

### Correlation Between IQGAP3 Expression and Infiltration of Immune Cells

The infiltration of immune cells has an indispensable role in cancer progression. Next, we explored the relationship between IQGAP3 expression and immune cell infiltration in different cancer types. The TIMER database showed that IQGAP3 expression was correlated significantly with the abundance of cluster of differentiation (CD)8+ T cells in 25 types of cancer, CD4^+^ T cells in 27 cancer types, neutrophils in 29 cancer types, dendritic cells in 30 cancer types, macrophages in 29 cancer types, and B cells in 29 cancer types (Supplementary Figure S16A).

To verify these results, we employed the xCell (https://xcell.ucsf.edu/) database to assess the correlation between IQGAP3 expression and immune cell infiltration in diverse cancer types. IQGAP3 expression was positively correlated with 38 types of immune cells in 27 cancer types and negatively correlated with 38 types of immune cells in one cancer type (Supplementary Figure S16B). These findings indicated that IQGAP3 expression was significantly correlated with the infiltration of immune cells in human cancer.

### Correlation Between IQGAP3 Expression and Immune Modulators

**FIGURE 11:**
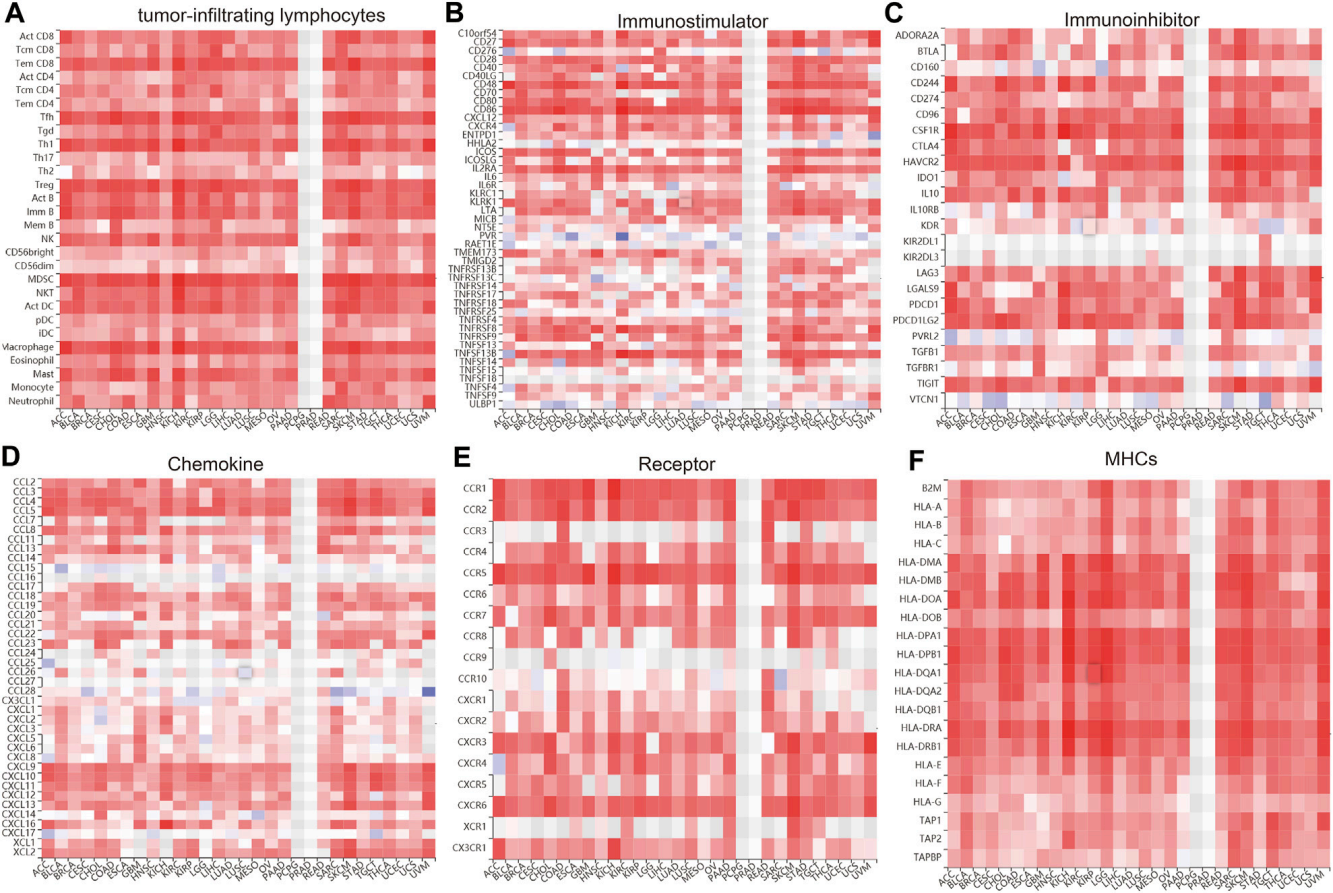
Analysis of the correlation between the IQGAP3 expression and diverse immune regulators. **(A)** The correlation between the IQGAP3 expression and 28 tumor-infiltrating lymphocytes was analyzed in pan-cancer by using the TISIDB database. **(B)** The correlation between the IQGAP3 expression and 45 immune stimulators in pan-cancer was examined by using the TISIDB database. **(C)** The correlation between the IQGAP3 expression and 24 immune inhibitors in pan-cancer was examined by using the TISIDB database. **(D)** The correlation between the IQGAP3 expression and 41 chemokines in pan-cancer was examined by using the TISIDB database. **(E)** The correlation between the IQGAP3 expression and 18 receptors in pan-cancer was examined by using the TISIDB database. **(F)** The correlation between the IQGAP3 expression and 21 MHCs in pan-cancer analysis was performed by using the TISIDB database.

We wished to further understand the relationship between IQGAP3 and the tumor microenvironment. We examined the correlation between IQGAP3 expression and immune checkpoint–related genes using the TCGA database. IQGAP3 expression was positively correlated with immune checkpoint–related genes in 31 cancer types. These immune checkpoint–related genes were CD274, CTLA4, HAVCR2, LAG3, PDCD1, PDCD1LG2, SIGLEC15, and TIGIT (Supplementary Figure S17). The TISIDB (http://cis.hku.hk/TISIDB/) database showed that IQGAP3 expression was positively correlated with 28 tumor-infiltrating lymphocytes, 45 immune stimulators, 24 immune inhibitors, 41 chemokines, 18 receptors, and 21 major histocompatibility complex (MHC) molecules in different cancer types (Figures 11A–F). These findings indicated that IQGAP3 had an indispensable role in the regulation of the immune response in human cancers.

### Correlation Between IQGAP3 Expression and Drug Sensitivity

The results detailed above suggested that IQGAP3 may have roles in cancer progression. Next, we explored the correlation between IQGAP3 expression and sensitivity to different drugs in different cancer cell lines from the GDSC database and Cancer Therapeutics Response Portal (CTRP) (https://portals.broadinstitute.org/ctrp/).

**FIGURE 12:**
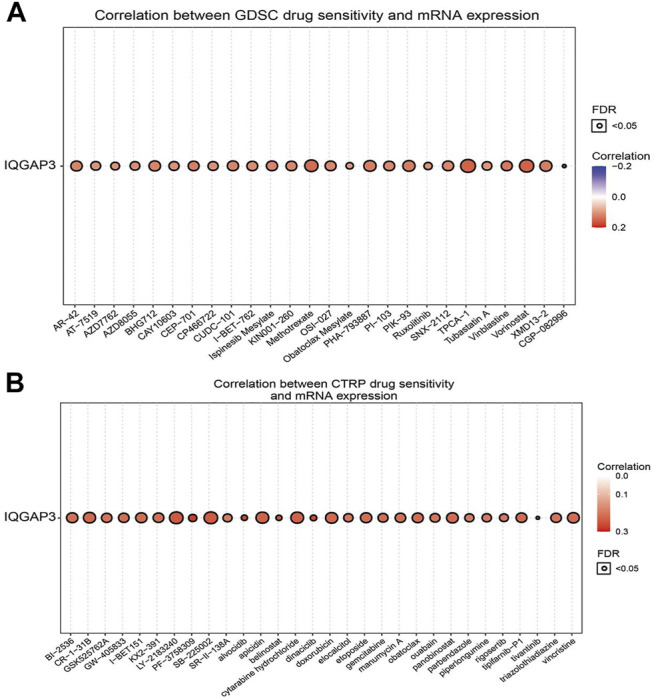
Analysis of the correlation between the IQGAP3 expression and drug sensitivity in diverse human cancers. **(A)** The correlation between the IQGAP3 expression and drug sensitivity in diverse human cancer analyses was performed by employing the GDSC database. **(B)** The correlation between the IQGAP3 expression and drug sensitivity in diverse human cancer analyses was performed by employing the CTRP database.

IQGAP3 expression was positively correlated with sensitivity to the drugs TPCA-1, vorinostat, methotrexate, PHA-793887, PIK-93, XMD13-2, BHG712, AR-42, CUDC-101, ispinesib mesylate, SNX-2112, OSI-027, and vinblastine in the GDSC database (Figure 12A and Supplementary Table S8). In the CTRP database, we observed IQGAP3 expression to be positively correlated with sensitivity to the 53 drugs shown in Figure 12B and Supplementary Table S9. In summary, these results demonstrated that IQGAP3 expression was significantly correlated with sensitivity to many drugs in different cancer cell lines.

### IQGAP3 Shows High Expression in NSCLC

**FIGURE 13:**
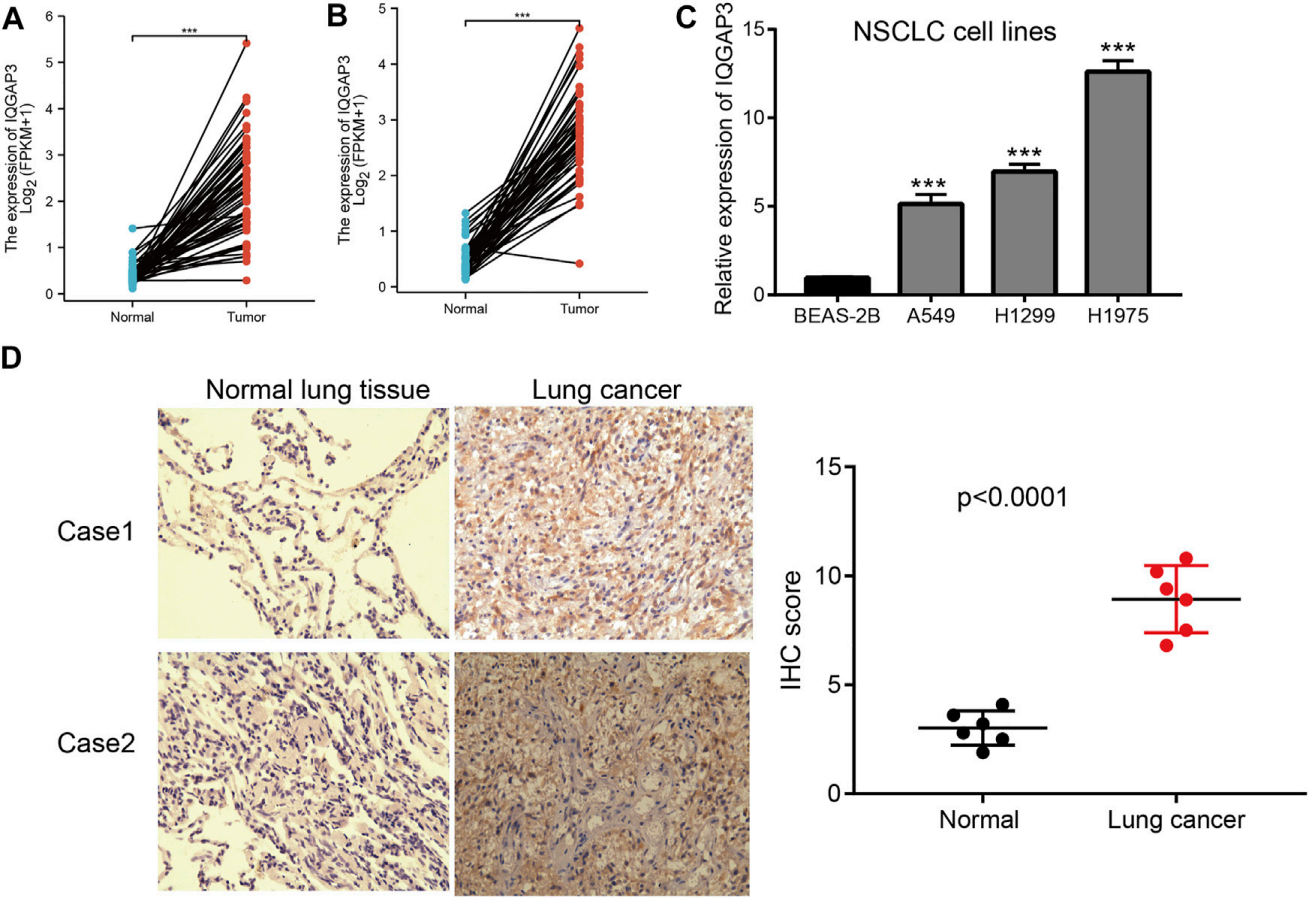
Analysis of the expression of IQGAP3 expression in NSCLC. **(A)** The expression of IQGAP3 in LUAD was examined by using the TCGA LUAD database. **(B)** The expression of IQGAP3 in LUAD was examined by using the TCGA LUAD database. **(C)** The expression of IQGAP3 in NSCLC cell lines was examined by using the qRT-PCR assay. **(D)** The expression of IQGAP3 in lung cancer was examined by using an IHC assay.

We showed that IQGAP3 expression was upregulated significantly in NSCLC using TGCG/LUAD/LUSC data. To verify this finding, we measured the mRNA and protein expressions of IQGAP3 in NSCLC. Real-time RT-qPCR and IHC assays showed that IQGAP3 expression was increased in NSCLC cell lines and lung cancer tissues compared with normal lung cells and normal lung tissues, respectively. These findings demonstrated that IQGAP3 expression was upregulated in NSCLC and indicated that IQGAP3 may have a crucial regulatory role in NSCLC progression (Figure 13).

## Discussion

Resection, radiotherapy, and adjuvant chemotherapy can be used to treat cancer, but their efficacy is limited (Siegel et al., 2021). Integrative genomics, transcriptomics, proteomics, metabolomics, and single-cell “omics” have been developed to unearth the biomarkers related to the occurrence and development of cancer.

Emerging evidence has shown that IQGAP3 participates in different cancer-related signaling pathways. IQGAP3, *via* interaction with protein kinase C (PKC)δ and the competitive inhibition of the interaction between PKCδ and PKCα, leads to PKCα phosphorylation, as well as activation and promotion of cell proliferation (Lin et al., 2019). Studies have suggested that E2F1 can activate IQGAP3 expression at the transcription level (Lin et al., 2019). However, whether lncRNAs and miRs regulate IQGAP3 expression at the post-transcriptional level is not known.

Using various public databases, we found that IQGAP3 expression was upregulated in different types of human cancer. High expression of IQGAP3 was correlated significantly with the tumor stage and lymph-node metastasis of different cancer types. High expression of IQGAP3 was also associated with a poor prognosis in some types of human cancer. These results suggested IQGAP3 to have a crucial role in the oncogenesis and tumor progression in humans. Analyses of DNA methylation in the promoter region of IQGAP3 revealed that a low level of DNA methylation of IQGAP3 was significantly negatively correlated with IQGAP3 expression in ACC, BLCA, BRCA, CHOL, COAD, KIRC, LGG, LIHC, LUAD, LUSC, PAAD, READ, SARC, SKCM, STAD, TGCT, THCA, UCEC, UCS, and UVM. Survival analyses showed that a low DNA-methylation level of IQGAP3 was correlated with the better poor prognosis in diverse cancer. Amplification was the main reason for the mRNA of IQGAP3 to be upregulated in human cancer.

Usually, transcription factors bind to the promoter region of a gene and regulate gene transcription. We found that PTBP1 (an RNA-binding protein) could be a transcription factor of IQGAP3 in human cancer. PTBP1 expression was significantly positively corelated with IQGAP3 expression in human cancer. In LIHC, using short hairpin-RNA knockdown of PTBP1, reduced IQGAP3 expression markedly. These results indicated that PTBP1 may be a potential transcription factor of IQGAP3.

IQGAP3 was involved mainly in angiogenesis, apoptosis, cell cycle, cell differentiation, DNA damage, EMT, hypoxia, inflammation, invasion, metastasis, and proliferation in human cancer. High expression of IQGAP3 was positively correlated with the cell cycle, cell proliferation, DNA damage, DNA repair, EMT, and inflammation in different cancer types. These findings suggested that IQGAP3 has a pivotal role in the initiation and prognosis of human cancer. The genes most closely associated with IQGAP3 were those for CDC42, MYL6B, MYH2, RAC1, PPP1R16A, MEF2A, IST1, NDC80, HIF1A, ITPRIPL2, CALM1, RAC3, RAC2, SRGAP3, KIFAP3, COG2, NPHP4, IQGAP1, IQGAP2, and CLUAP1. The proteins most closely associated with IQGAP3 were CDC42, IQGAP2, KIF20, CDH1, MEN1, CTNNB1, CTNNA1, IQGAP1, RAC1, and CLIP1. These proteins have been reported to have crucial roles in the cell proliferation and cell cycle of diverse cancer types (Maldonado and Dharmawardhane, 2018).

TMB and MSI have emerged as specific and sensitive biomarkers of the response to immune-checkpoint inhibitors (Ghandi et al., 2019). We found that IQGAP3 expression was significantly associated with TMB and MSI in diverse cancer types. Our findings on the link between IQGAP3 expression and the abundance of immune cells, expression of immune checkpoint-related genes, and expression of proinflammatory moieties indicated that IQGAP3 has an indispensable role in regulation of the immune response in human cancer.

Let-7c-5p showed low expression in CHOL, BRCA, BLCA, UCEC, THCA, STAD, LUSC, LUAD, LIHC, KICH, HNSC, and COAD, and low expression of let-7c-5p correlated with the tumor stage in different cancer types. High expression of let-7c-5p not only correlated with a good prognosis in BRCA, CECS, ESCA, HNSC, KIRP, LIHC, LUAD, and LUSC, but also correlated with a poor prognosis in BLCA, PAAD, and STAD. IQGAP3AR expression was significantly negatively correlated with let-7c-5p expression. IQGAP3 expression was positively correlated with sensitivity to different drugs in different cancer cell lines. In total, we provided the first evidence that a IQGAP3AR/let-7c-5p/IQGAP3 axis has indispensable roles in the progression and immune response to different types of human cancer.

## Conclusions

This is the first study to characterize the expression, prognosis, DNA methylation, and gene mutation of IQGAP3 in different types of human cancer. We showed that IQGAP3 expression was positively correlated with TMB, MSI, immune cell infiltration, and immune modulators in diverse human cancers. Collectively, our findings revealed that the IQGAP3AR/let-7c-5p axis–mediated upregulation of IQGAP3 expression promoted cancer progression and immune cell infiltration in different types of human cancer. The IQGAP3AR/let-7c-5p axis could be a diagnostic and therapeutic biomarker for cancers.

## Data Availability

The original contributions presented in the study are included in the article/Supplementary Material, further inquiries can be directed to the corresponding author.
