# Supplementary material for: Comprehensive Analyses of the Immunological and Prognostic Roles of an IQGAP3AR/let-7c-5p/IQGAP3 Axis in Different Types of Human Cancer
Source: Front Mol Biosci. 2022 Feb 22;9:763248. doi: 10.3389/fmolb.2022.763248 (PMC8902246; doi:10.3389/fmolb.2022.763248)
Supplement: Supplementary file 1 [file DataSheet1.docx]

Supplementary Material

**Supplementary FIGURE 1⎜** **Analysis of the tumor stage for IQGAP3 in human cancers.**

**Analysis the tumor stage for IQGAP3 in PAAD, BRCA and LIHC (A), LUAD, LUSC and OV (B) by GEPIA database.**

**
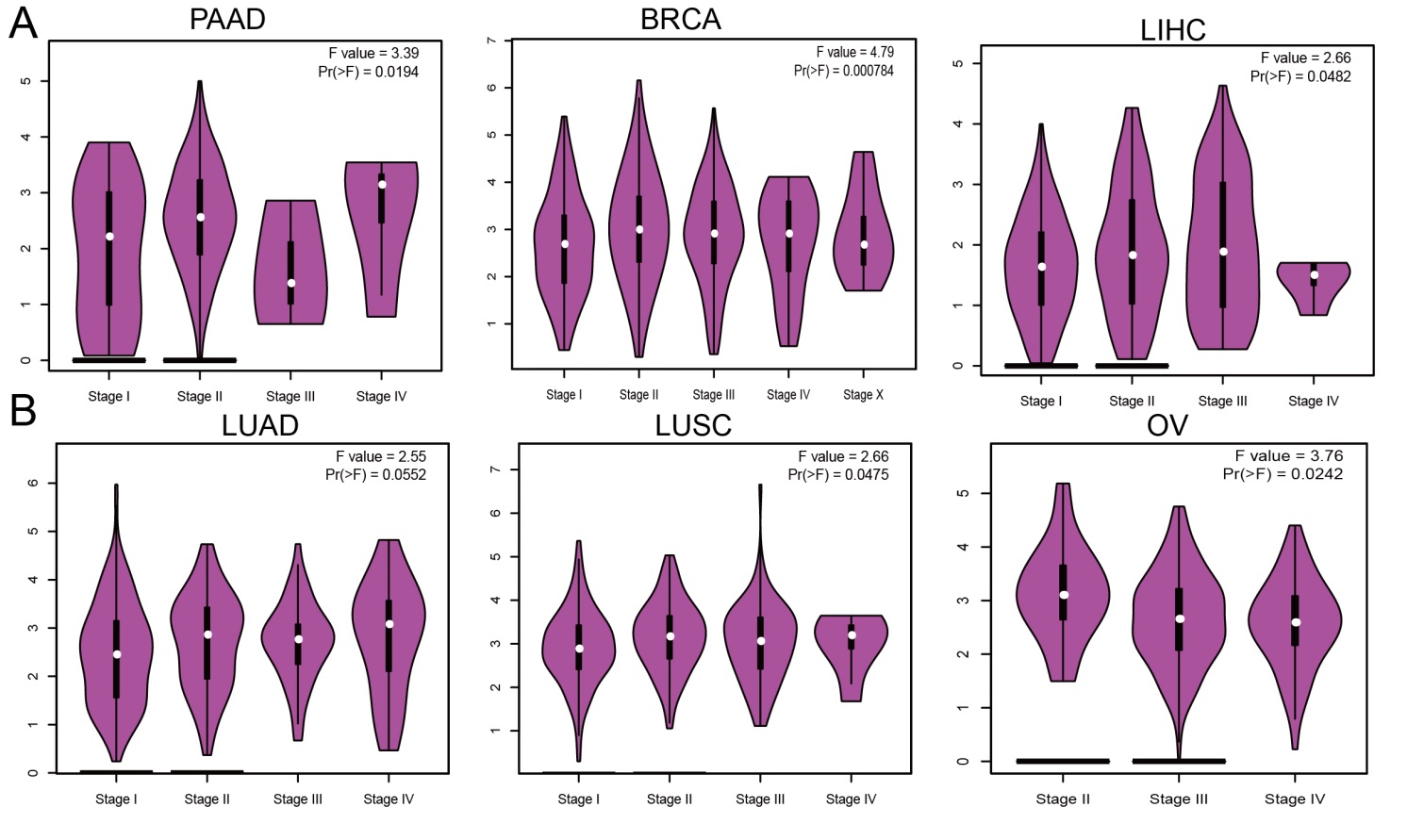
**

**Supplementary FIGURE 2⎜ Association between the IQGAP3 expression and lymph node metastasis of pan-cancer.** The correlation between the IQGAP3 expression and Lymph node metastasis in CESC, BLCA, THCA and READ (A), PRAD, LUSC, LUAD and KIRP (B), KICH, HNSC , COAD and CHOL (C) analysis by the UALCAN database.

**
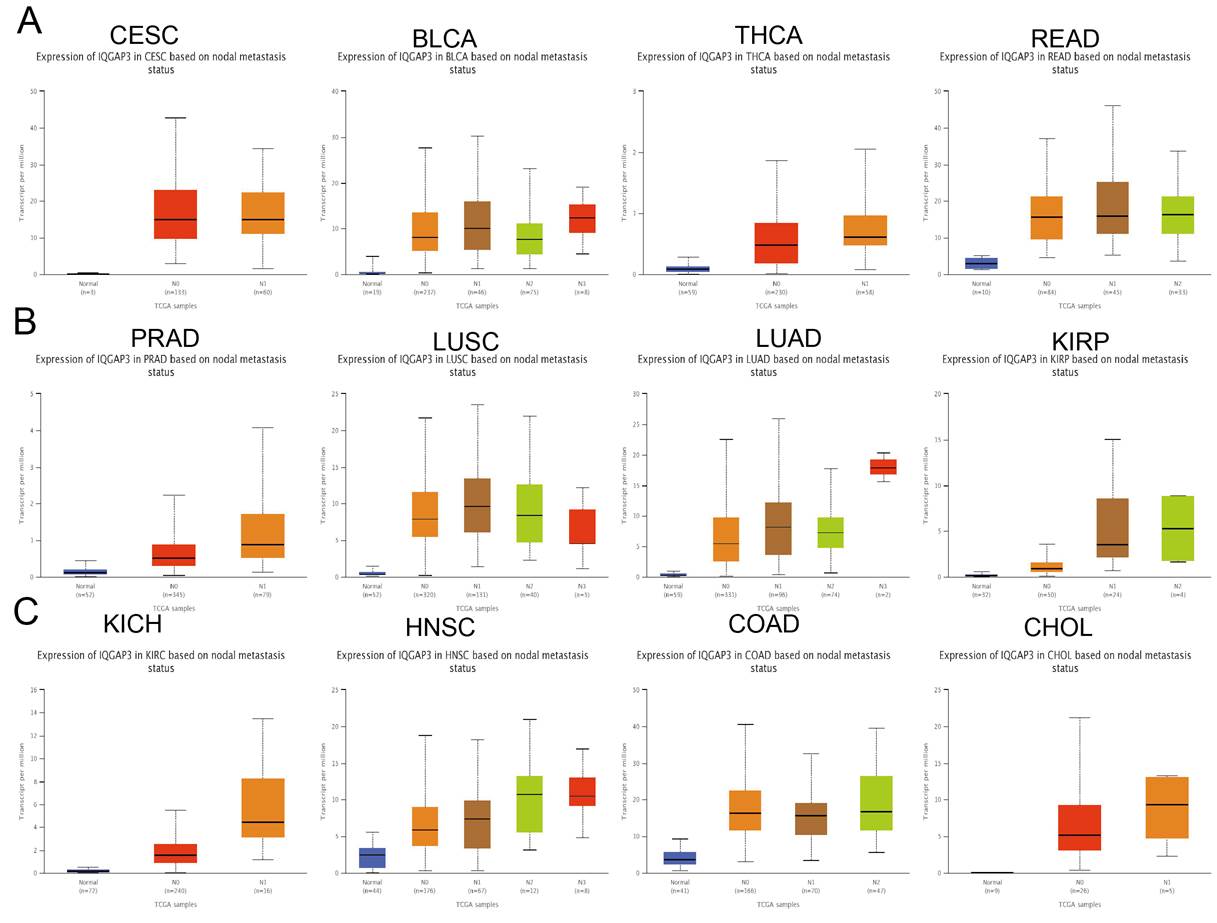
**

**Supplementary FIGURE 3⎜** **Analysis of the overall survival of IQGAP3 in human cancers.**

**The overall survival for IQGAP3 in LAML, KIRC, and UVM (A), LUAD (B) analysis by GEPIA database.**

**
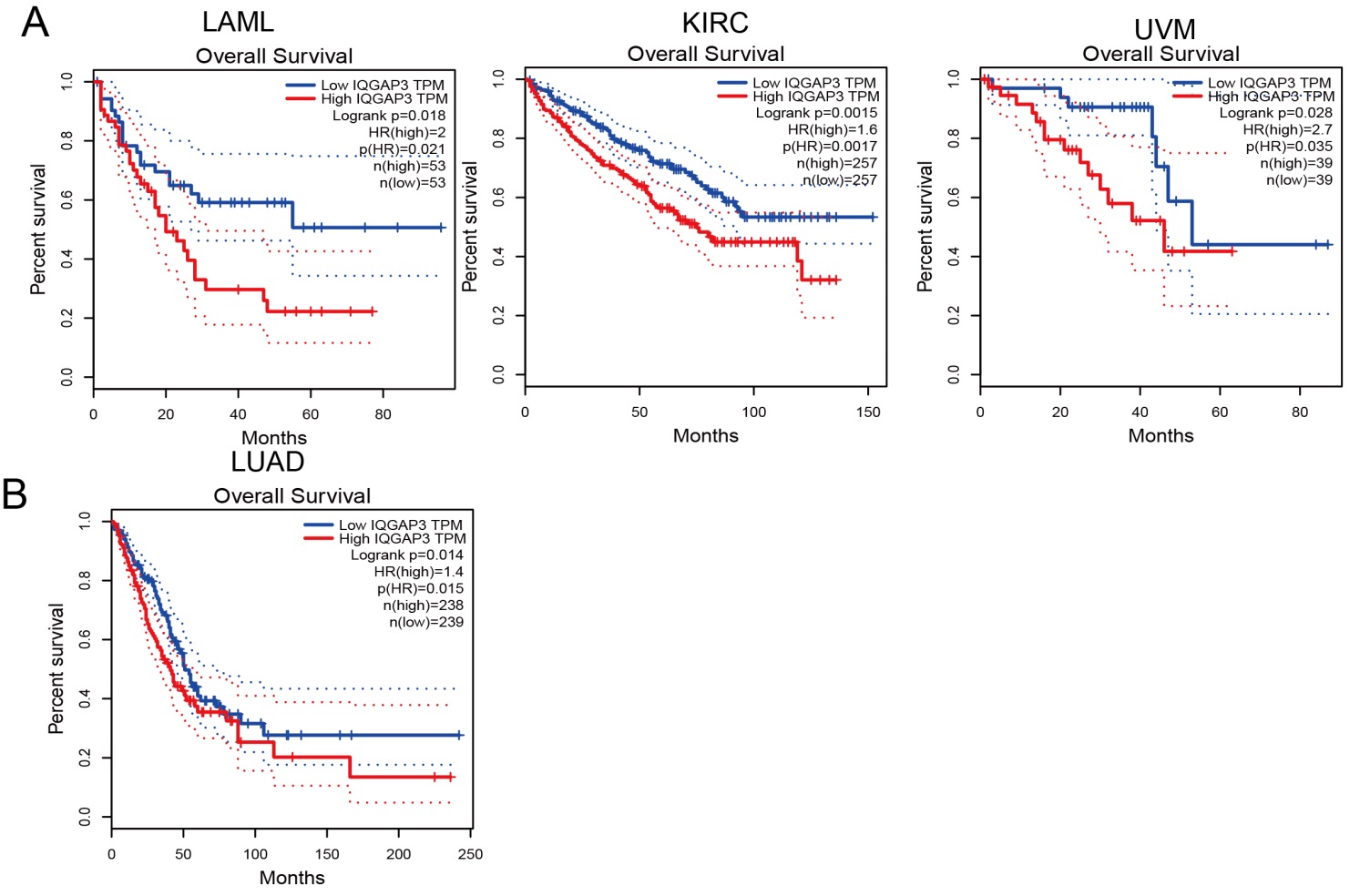
**

**Supplementary FIGURE 4 ⎜ Association between IQGAP3 expression and the OS, PFS, DFS and DSS of cancer patients.** (A) A forest plot of hazard ratios shown that the OS of IQGAP3 in 33 types of tumors. (B) A forest plot of hazard ratios shown that the PFS of IQGAP3 in 33 types of tumors. (C) A forest plot of hazard ratios shown that the DFS of IQGAP3 in 33 types of tumors. (D) A forest plot of hazard ratios shown that the DSS of IQGAP3 in 33 types of tumors.


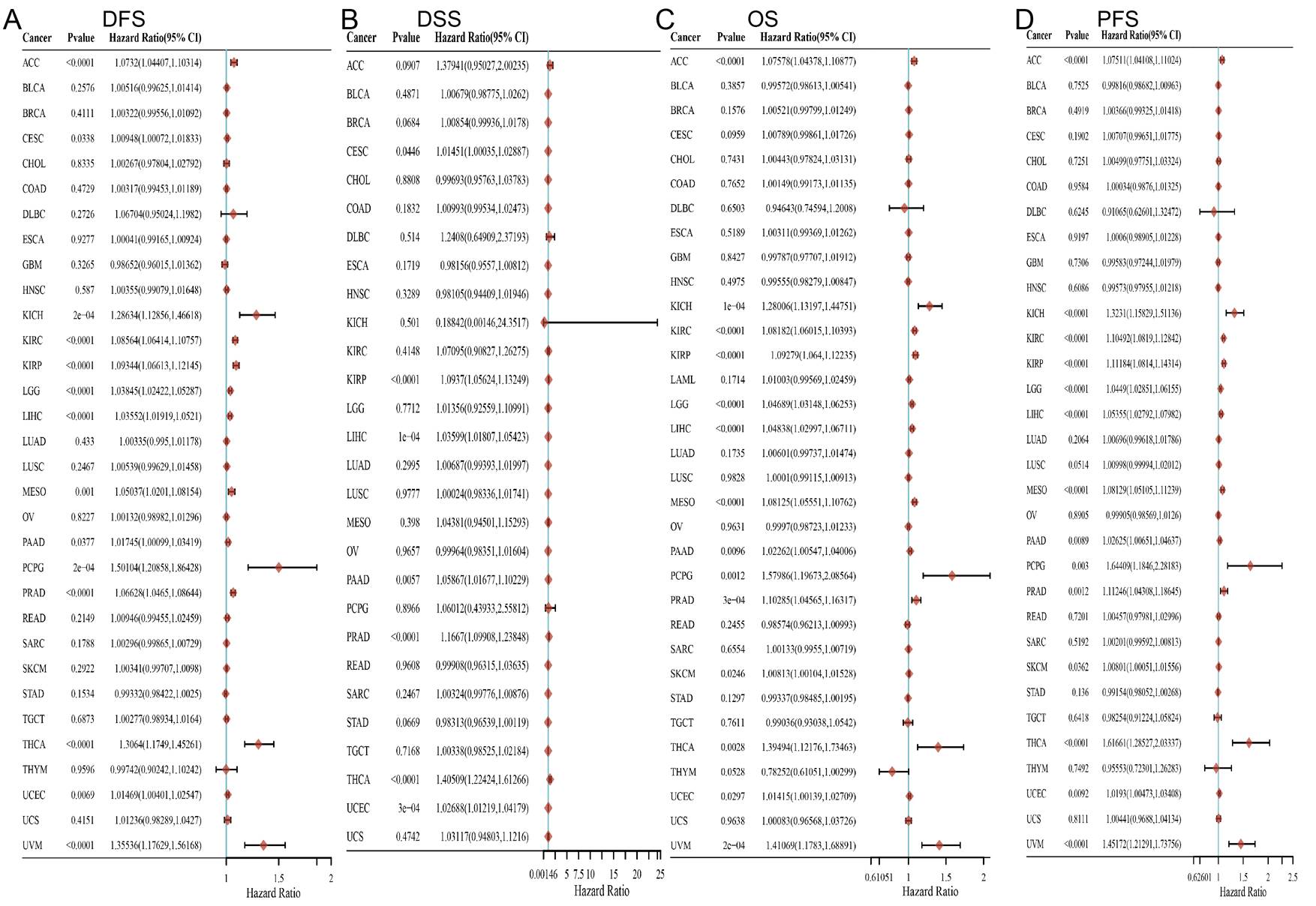


**Supplementary FIGURE 5 ⎜ Analysis of the diagnosis of IQGAP3 in diverse cancer.** (A) ROC curve analyses and AUC values for IQGAP3 in BRCA, PRAD, LUSC and LUAD. (B) ROC curve analyses and AUC values for IQGAP3 in KIRP, KIRC, COAD and RAED. (C) ROC curve analyses and AUC values for IQGAP3 in THCA, GBM, LGG and PAAD. (D) ROC curve analyses and AUC values for IQGAP3 in SKCM, LIHC, STAD and ESCA.


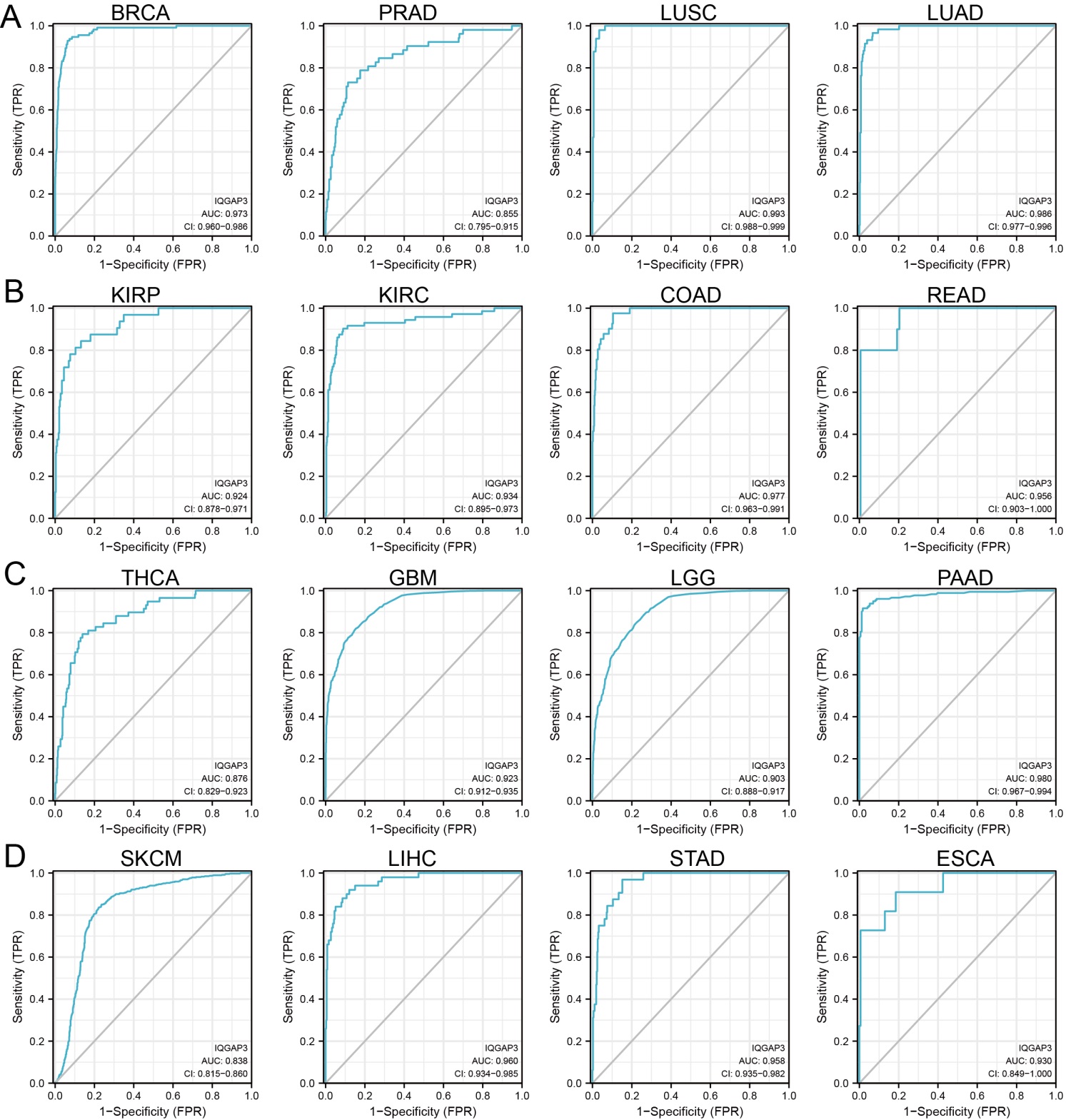


**Supplementary FIGURE 6 ⎜Analysis of the correlation between IQGAP3 expression and immune subtypes in pan-cancers.** (A-E) The expression of IQGAP3 in immune subtypes of BLCA, COAD, CHOL and CESC (A), KIRC, KICH, HNSC and ESCA (B), LUAD, LIHC, LGG and LUSC (C), MESO, PCPG, PAAD and READ (D), UCEC, STAD, SKCM and SARC (E).

**
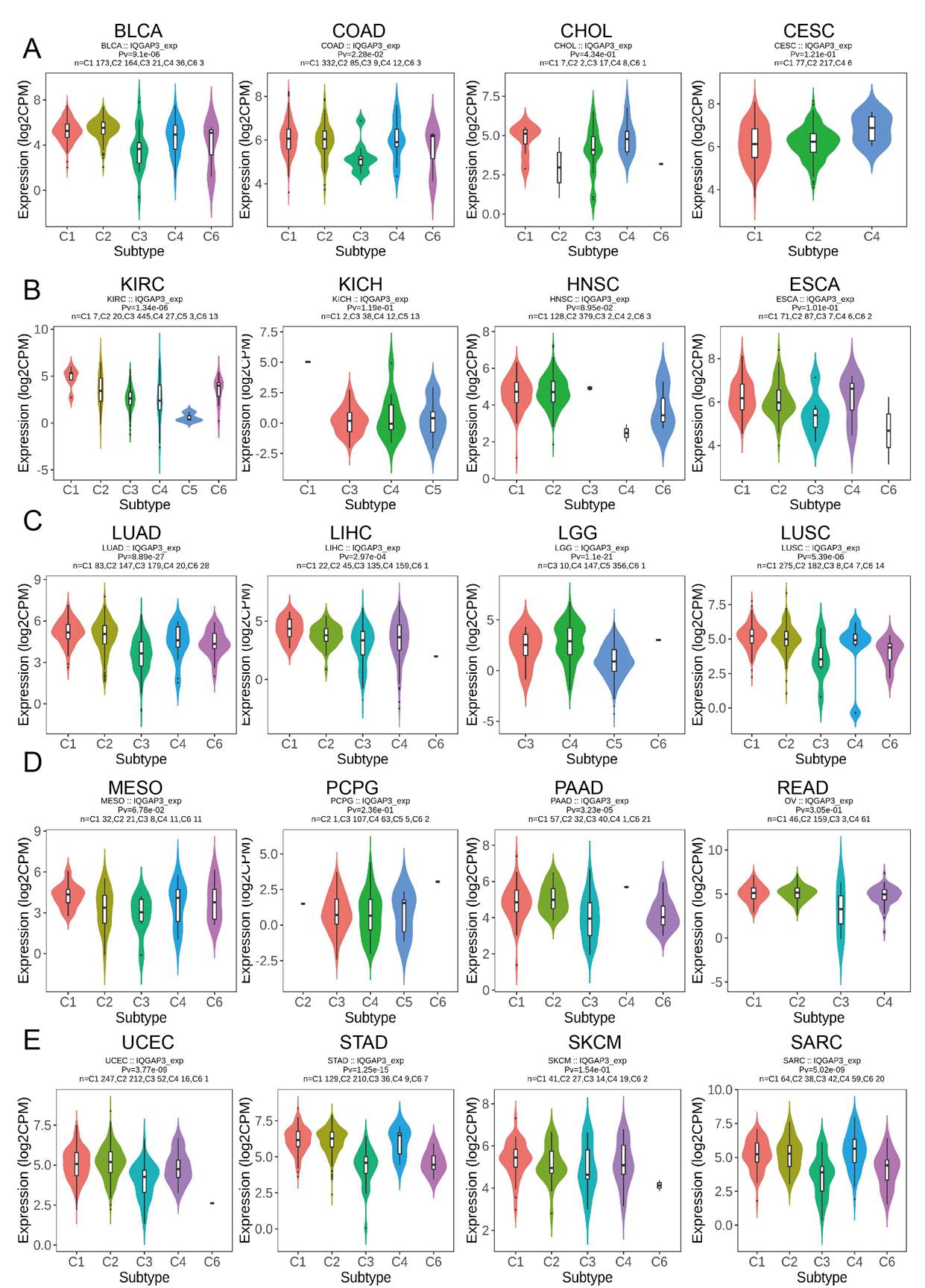
Supplementary FIGURE 7 ⎜Analysis of the relationship between IQGAP3 expression and molecular subtypes in pan-cancer.(A-C)** The expression of IQGAP3 in molecular subtypes of UCEC, STAD, SKCM and READ (A), OV, LUSC, LIHC and LGG (B), HNSC, COAD, BRCA and ACC (C).

**
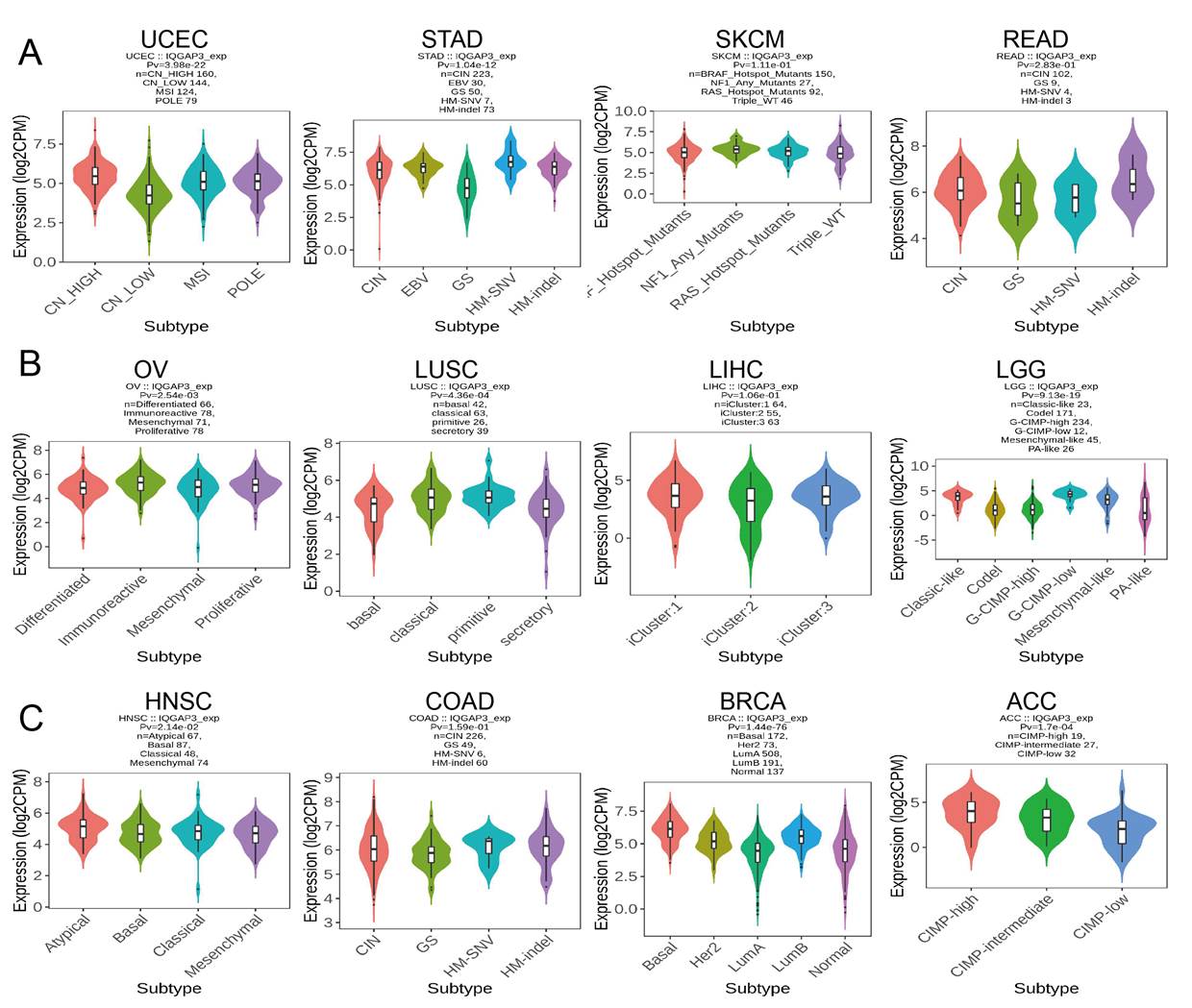
**

**Supplementary FIGURE 8** **⎜Correlation between PTBP1 and IQGAP3 expression in human pan-cancers.**

(A-D) Analysis the correlation between the PTBP1 and IQGAP3 in DLBC, ACC, UVM and THYM (A), TGCT, STAD, READ and PRAD (B), PAAD, MESO, LUSC and LUAD (C), LIHC, KICH, HNSC and LGG (D) by using the starbase tools. (E)The IQGAP3 expression after knock down of PTBP1 examined by using the GEO data.

**
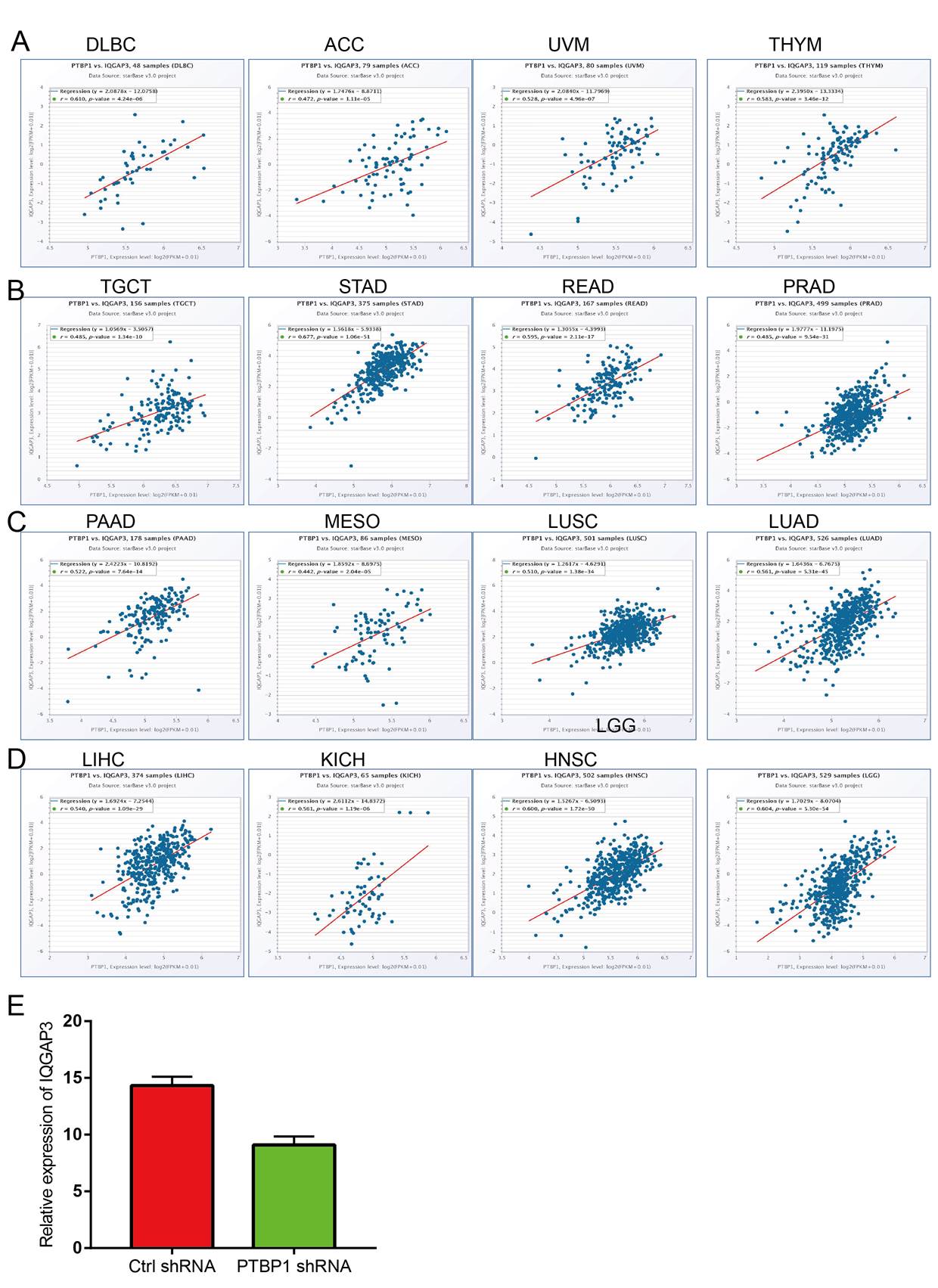
**

**Supplementary FIGURE 9 ⎜ The prognosis of PTBP1 in pan-cancer.**

(A-D)The prognosis of PTBP1 in BLCA, ESCA and ESCC (A), KIRP, LIHC and LUAD (B),

OV, PAAD and READ (C), SRAC, STAD and THYM (D) examine by the kmplot database.


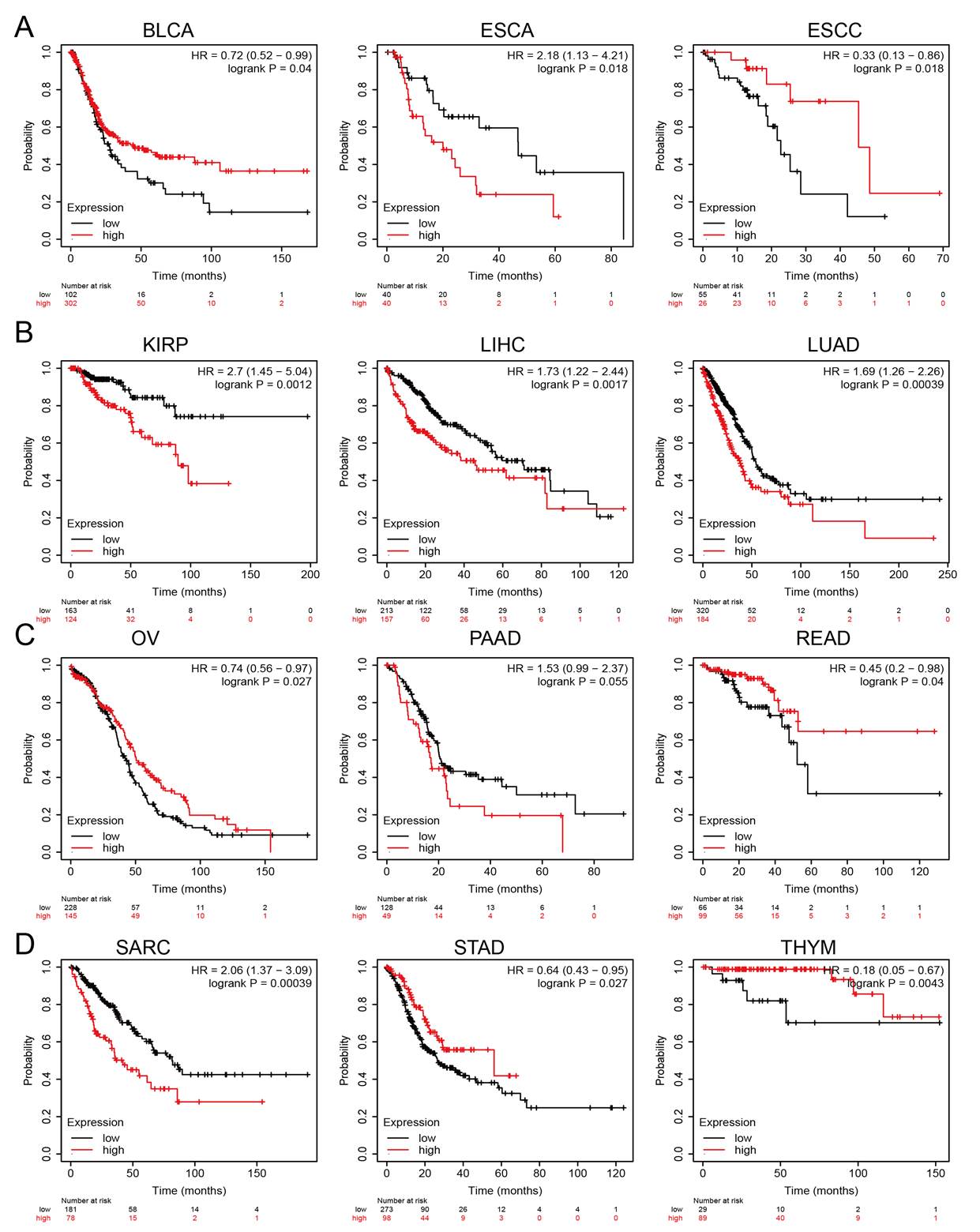


**Supplementary FIGURE 10 ⎜Analysis of the expression levels of let-7c-5p in pan-cancer.**

The expression of let-7c-5p in CHOL,BRCA, BLCA and UCEC (A),THCA, STAD, LUAD and LUSC (B), LIHC, KICH, HNSC and COAD (C) analysis by starbase database.

**
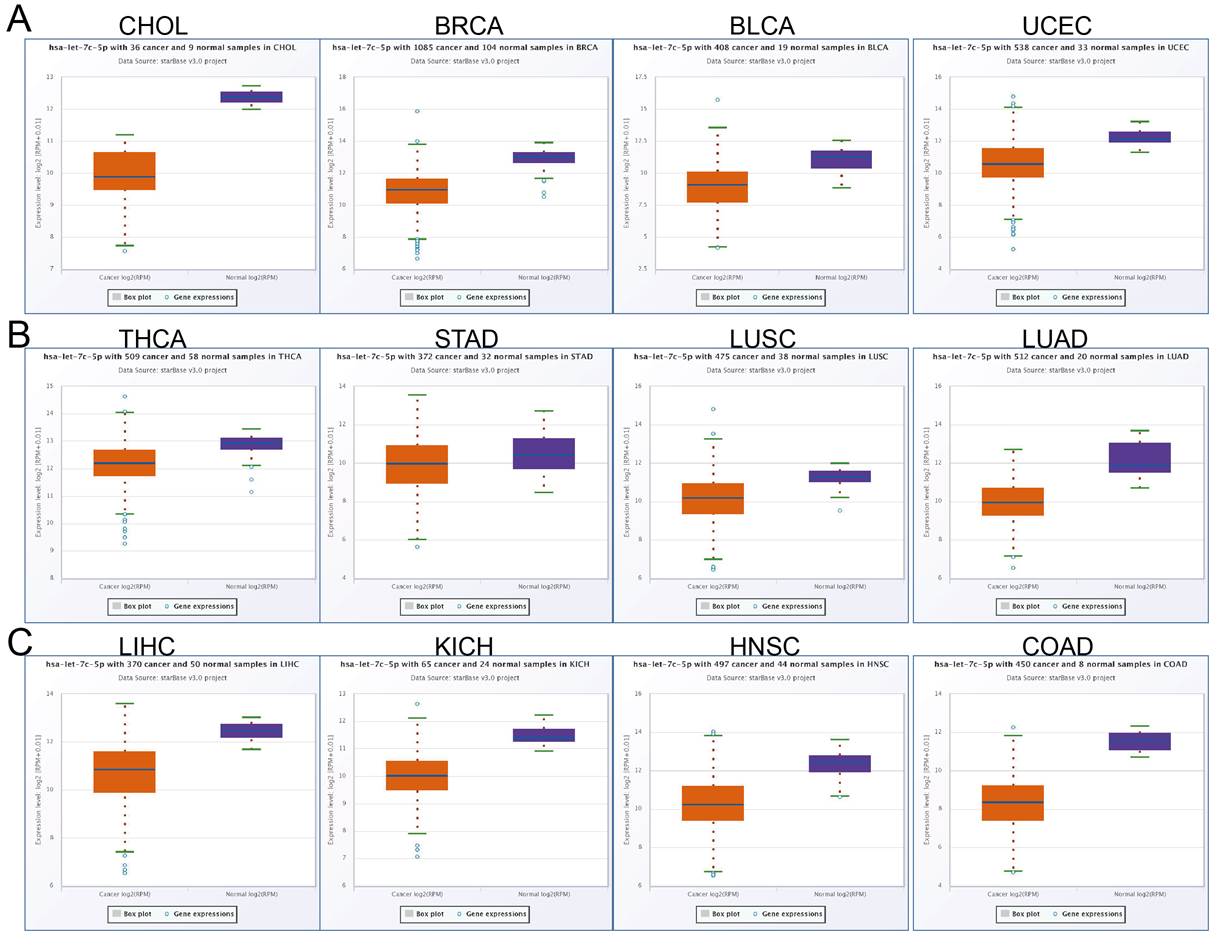
**

**Supplementary FIGURE 11⎜ The tumor stage of let-7c-5p in diverse cancer.**

(A-C) The correlation between the IQGAP3 expression and tumor stage in ACC, BLCA, BRCA and CESC (A), CHOL, COAD, HNSC and KIRP (B), LIHC, LUAD and LUSC (C) analysis by the UALCAN database.


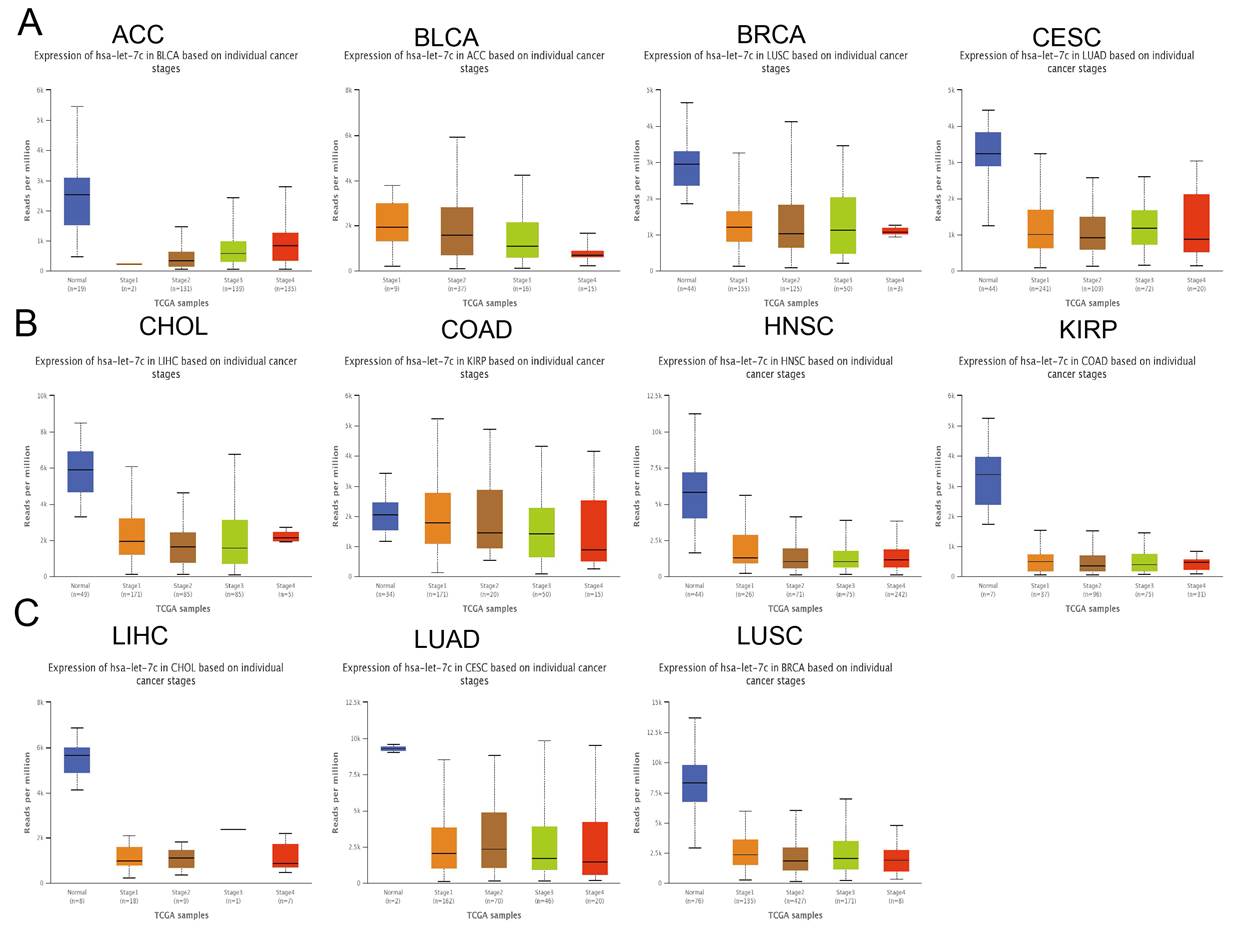


**Supplementary FIGURE 12 ⎜ Analysis of the prognosis of let-7c-5p in pan-cancer.**

The prognosis of let-7c-5p in BLCA, BRCA and CESC (A), ESCA, HNSC and KIRP (B),LIHC, LUAD and LUSC(C),PAAD and STAD(D) analysis by KMplot database.


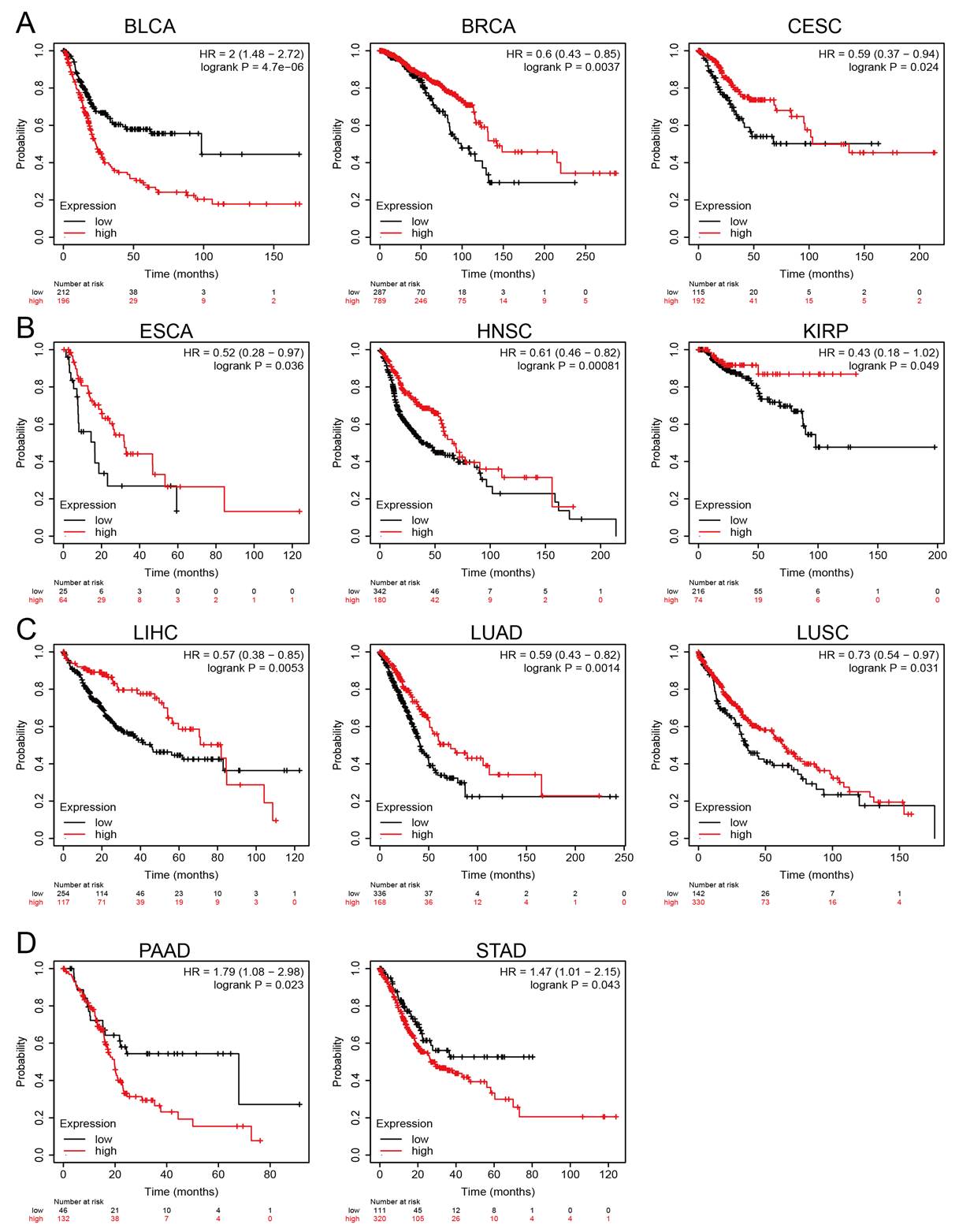


**Supplementary FIGURE 13 ⎜Analysis of the expression levels of IQGAP3AR in pan-cancer.**

The expression of IQGAP3AR in BRCA, BLCA, UCEC and STAD (A),PRAD, LUSC, LUAD and LIHC (B), KIRP, CHOL, KICH and HNSC (C),ESCA and COAD (D) analysis by starbase database.

(E) The relationship between diverse drug and lncRNA expression.


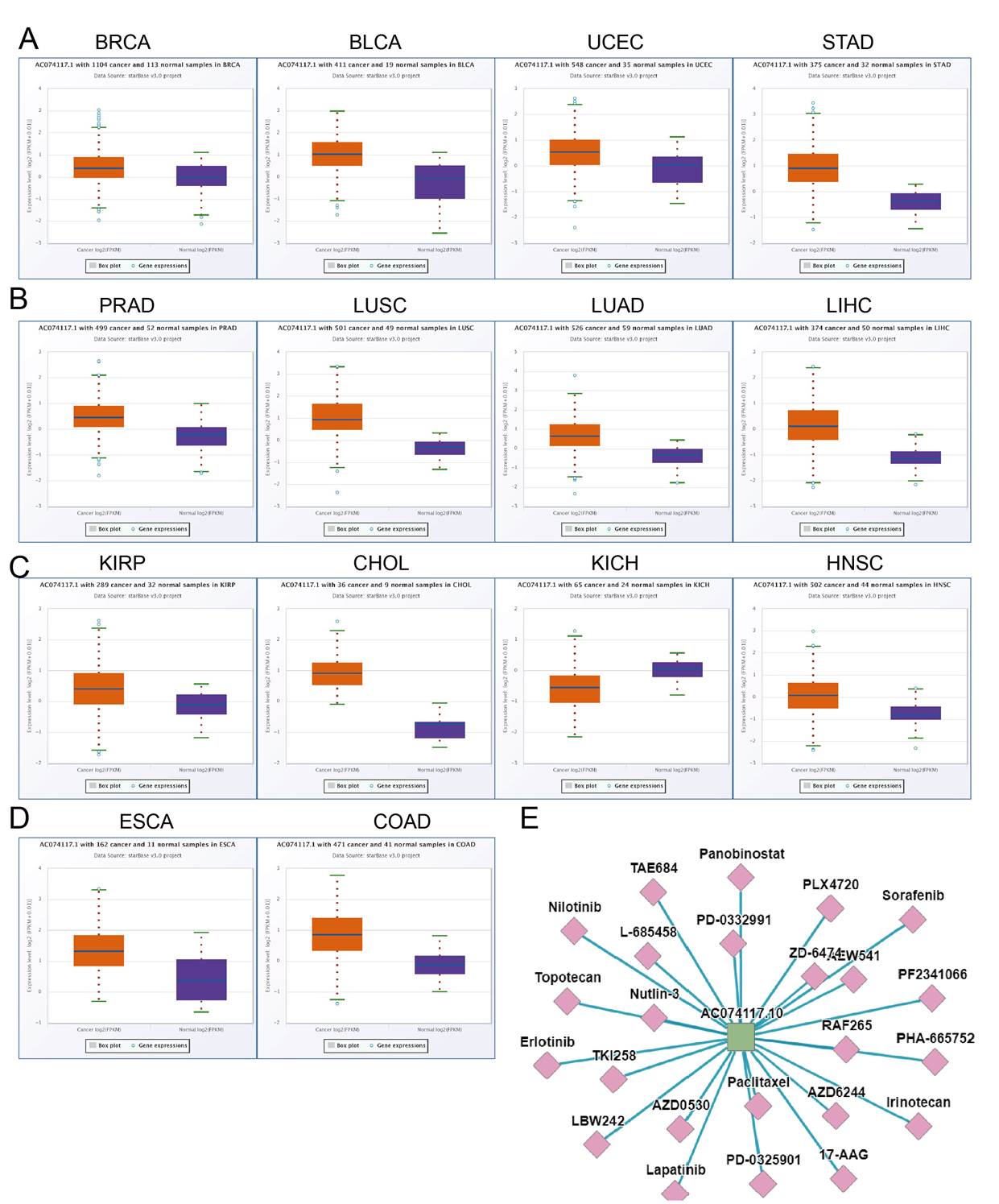


**Supplementary FIGURE 14 ⎜ The correlation between the IQGAP3AR expression and let-7c-5p in pan-cancer.** The correlation between the IQGAP3AR expression and let-7c-5p in BLCA, ACC, UCS and THYM (A), TGCT, PRAD, LUAD and LIHC (B), LGG, KIRP, DLBC and BRCA (C) analysis by using the starbase database.


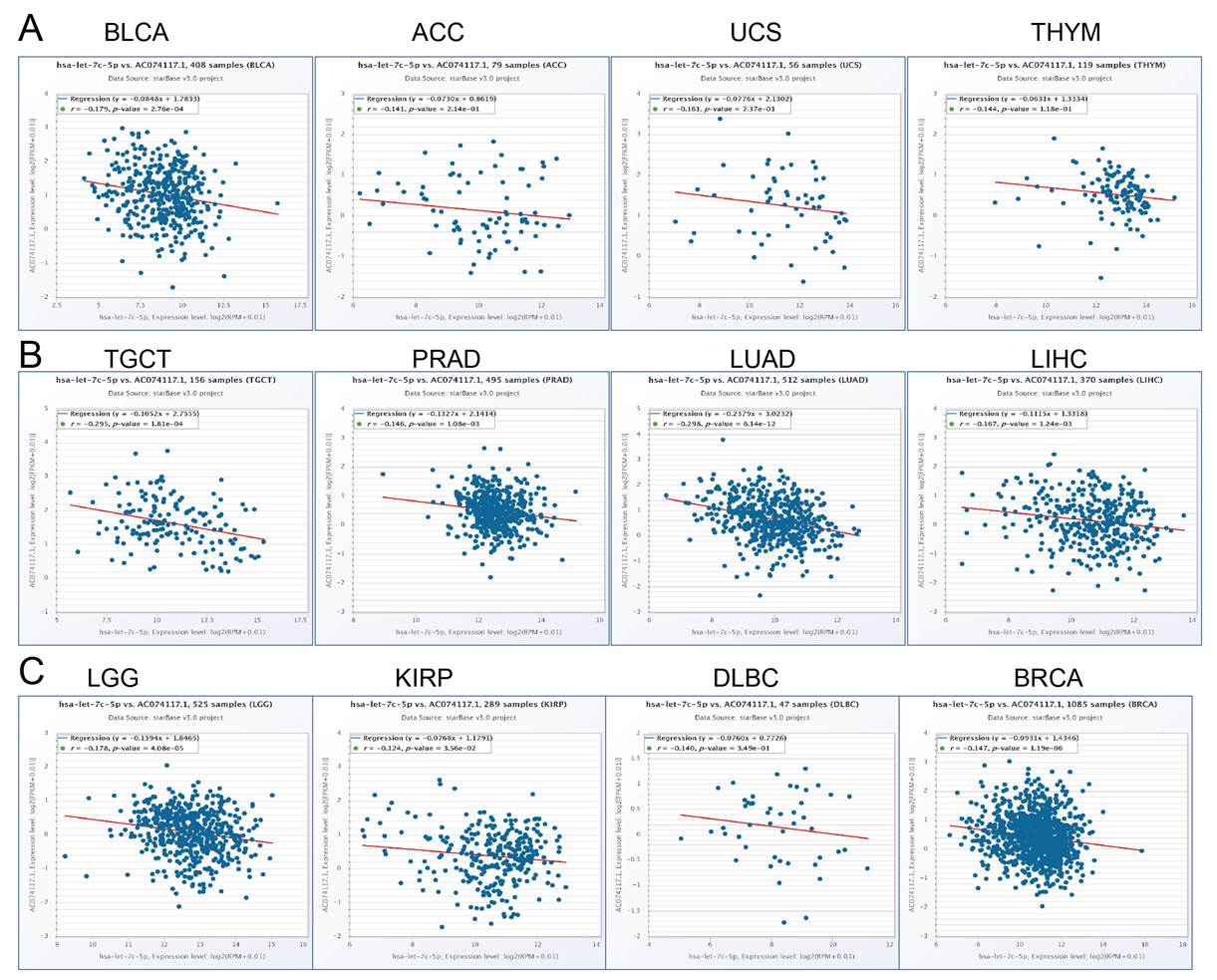


**Supplementary FIGURE 15 ⎜ The correlation between the IQGAP3AR expression and IQGAP3 in pan-cancer.**

The correlation between the IQGAP3AR expression and IQGAP3 in CESC, ACC, THY, and TGCT (A), STAD, PRAD, LUSC and LUAD (B), LGG, KIRP, KIRC and DLBC (C) analysis by using the starbase database.


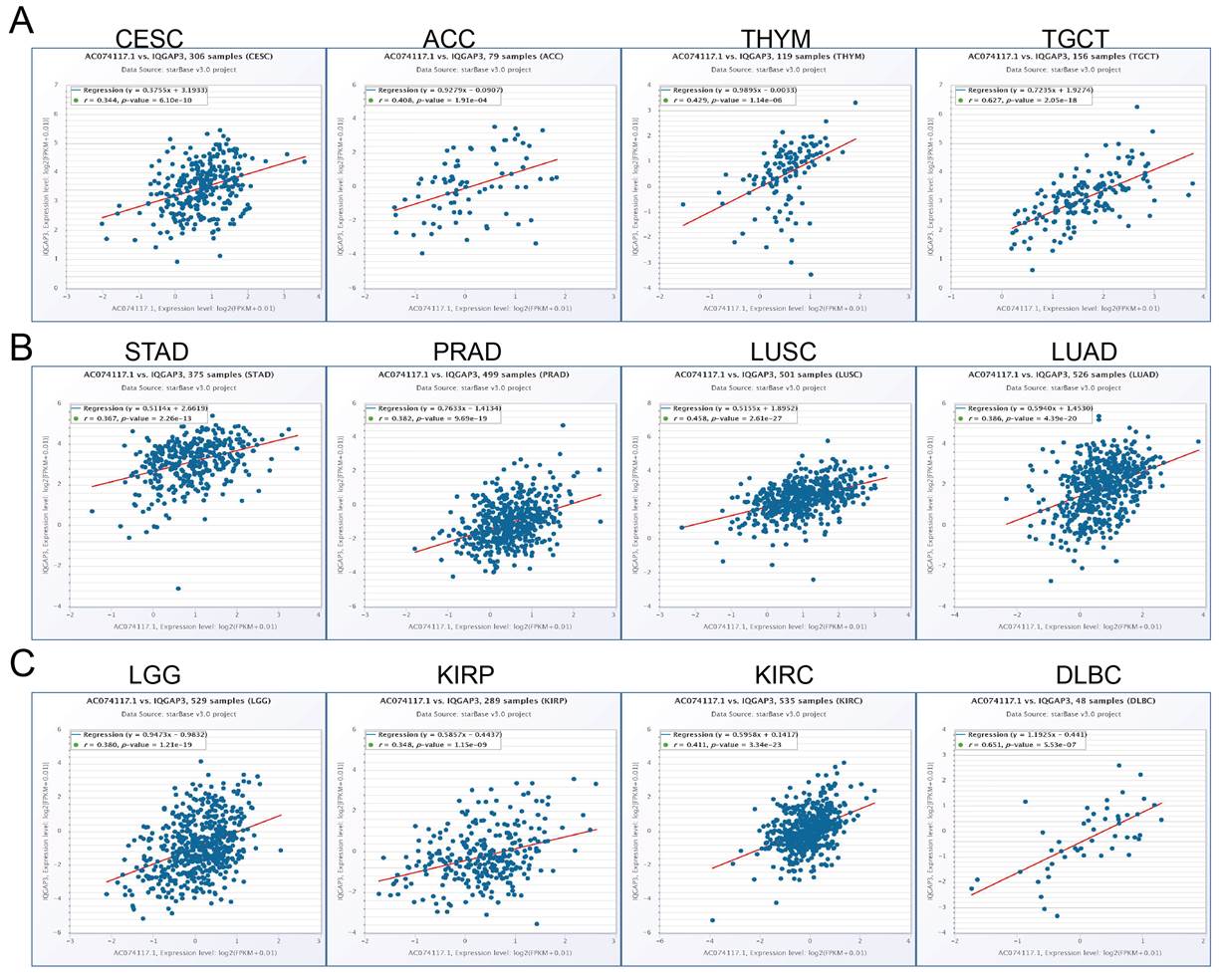


**Supplementary FIGURE 16 ⎜ Analysis the correlation between the IQGAP3 expression and immune cells infiltration.**

1. The correlation between the IQGAP3 expression and immune cells infiltration in pan-cancer analysis by the TIMER database.
2. The correlation between the IQGAP3 expression and diverse immune cells infiltration in pan-cancer analysis by the Xcell database.


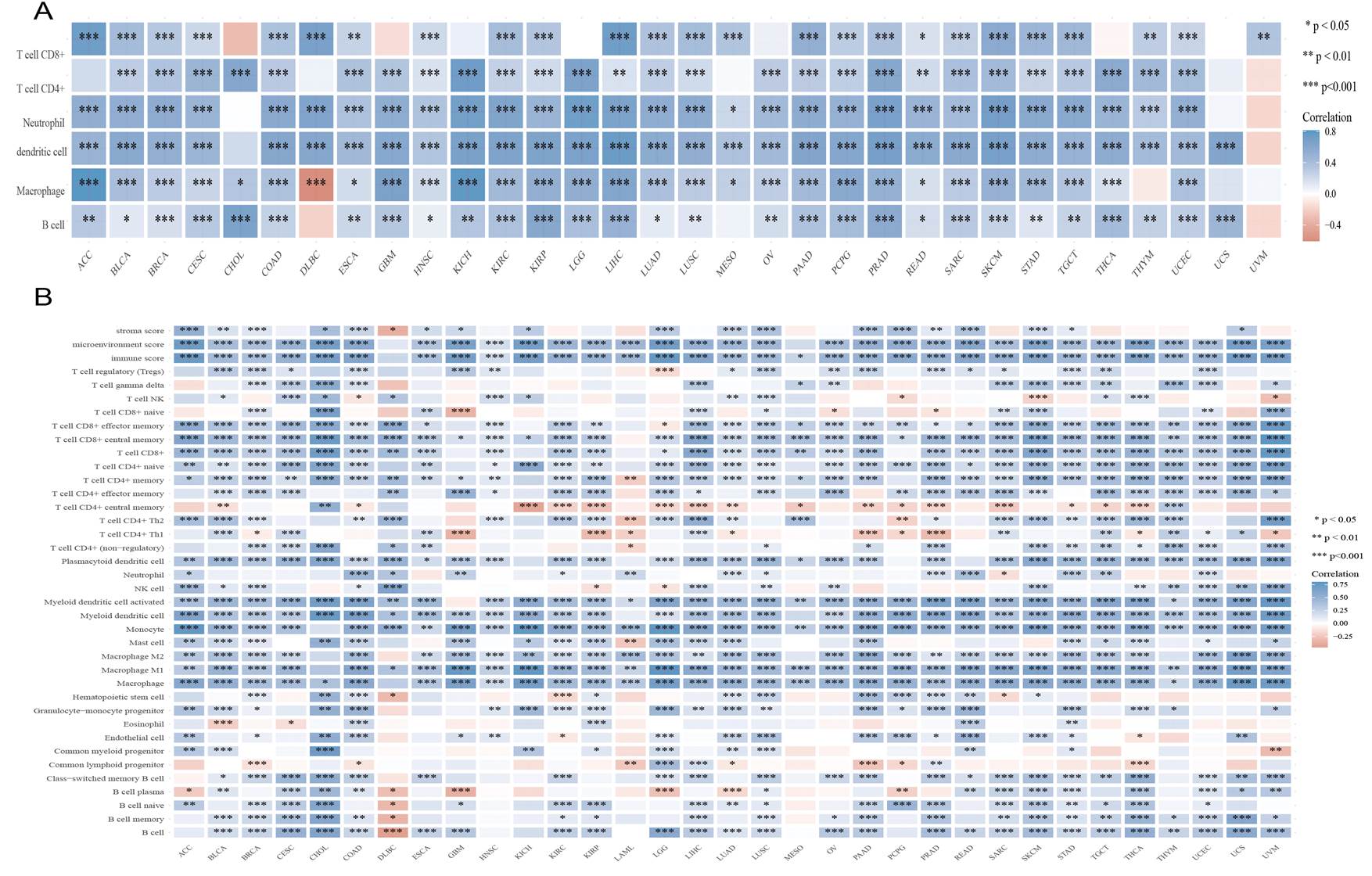


**Supplementary FIGURE 17 ⎜Analysis the correlation between the IQGAP3 expression and immune check points related gene.**

Analysis the correlation between the IQGAP3 expression and immune check points related gene in pan-cancer analysis by TIMER database.

**
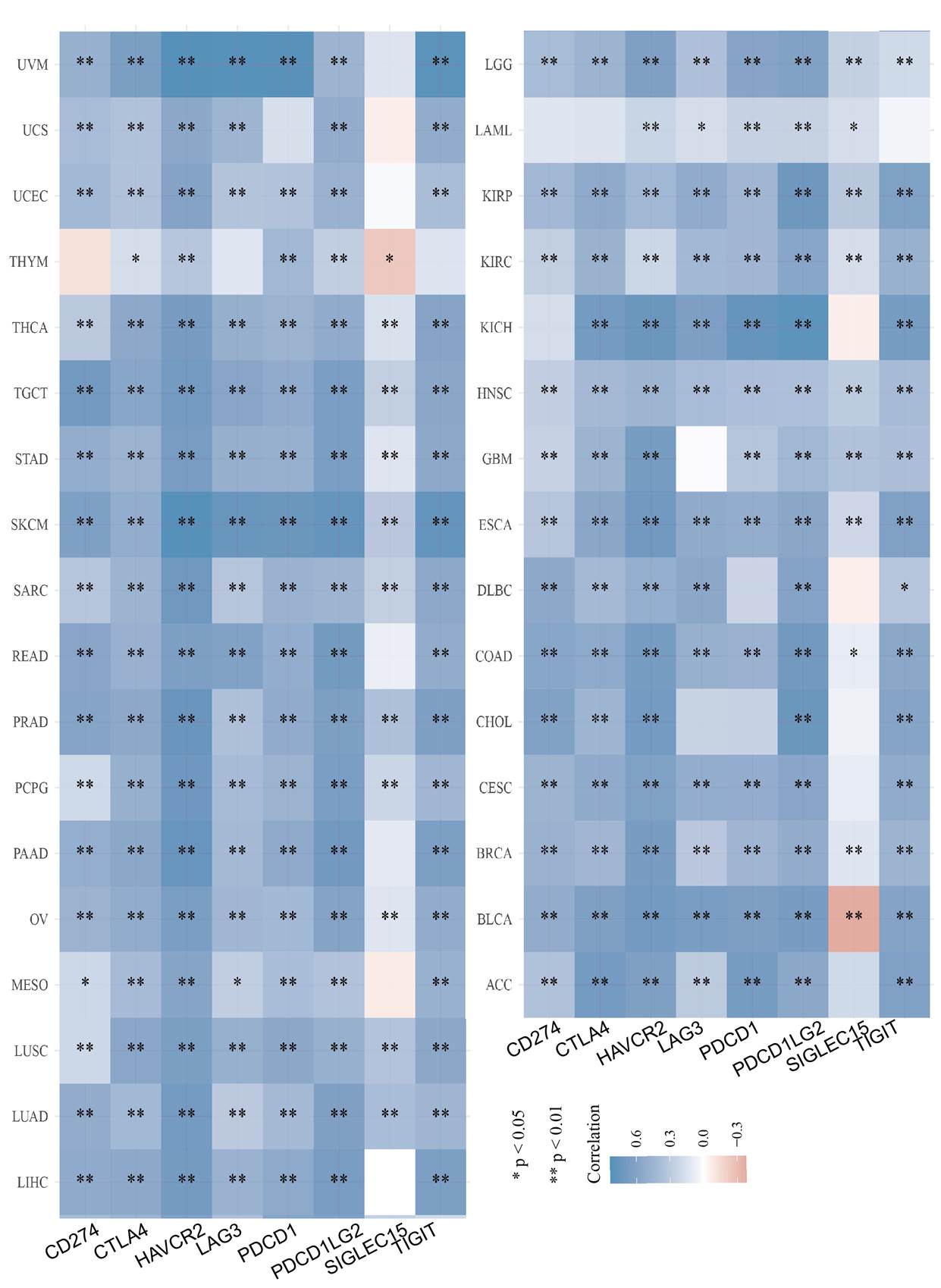
**

**⎜**

**Supplementary Table 1. The correlation between the CNV and IQGAP3 expression**

| IQGAP3 expression and CNV | | |
| --- | --- | --- |
| cancer type | r | p-value |
| ACC | 0.425 | *** |
| BLCA | 0.471 | *** |
| BRCA | 0.323 | *** |
| CHOL | 0.232 | *** |
| COAD | 0.288 | *** |
| KIRC | 0.212 | *** |
| LGG | 0.156 | *** |
| LIHC | 0.382 | *** |
| LUAD | 0.206 | *** |
| LUSC | 0.393 | *** |
| PAAD | 0.469 | *** |
| READ | 0.281 | *** |
| SARC | 0.446 | *** |
| SKCM | 0.389 | *** |
| STAD | 0.354 | *** |
| TGCT | 0.313 | *** |
| THCA | 0.313 | *** |
| UCEC | 0.285 | *** |
| UCS | 0.449 | *** |
| UVM | 0.23 | *** |

*P < 0.05, **P < 0.01, ***P < 0.001,

**Supplementary Table 2. The correlation between DNA methylation and IQGAP3 expression in human cancers**

| IQGAP3 expression and DNA methylation | | |
| --- | --- | --- |
| cancer type | r | p-value |
| ACC | -0.471 | *** |
| BLCA | -0.271 | *** |
| BRCA | -0.248 | *** |
| CHOL | -0.406 | *** |
| COAD | -0.21 | *** |
| KIRC | -0.293 | *** |
| LGG | -0.237 | *** |
| LIHC | -0.31 | *** |
| LUAD | -0.16 | *** |
| LUSC | -0.212 | *** |
| PAAD | -0.306 | *** |
| READ | -0.28 | *** |
| SARC | -0.317 | *** |
| SKCM | -0.342 | *** |
| STAD | -0.151 | *** |
| TGCT | -0.458 | *** |
| THCA | -0.334 | *** |
| UCEC | -0.183 | *** |
| UCS | -0.386 | *** |
| UVM | -0.319 | *** |

*P < 0.05, **P < 0.01, ***P < 0.001,

**Supplementary Table 3. The expression of let-7c-5p in pan-cancer analysis by the starbase database**

| **Cancer Type** | **CancerNum** | **NormalNum** | **CancerExp** | **NormalExp** | **FoldChange** | **pValue** |
| --- | --- | --- | --- | --- | --- | --- |
| [BLCA](http://starbase.sysu.edu.cn/panMirDiffExp.php) | 408 | 19 | 962.09 | 2706.53 | 0.36 | 1.80E-08 |
| [BRCA](http://starbase.sysu.edu.cn/panMirDiffExp.php) | 1085 | 104 | 2407.87 | 8034.34 | 0.3 | 1.10E-63 |
| [HNSC](http://starbase.sysu.edu.cn/panMirDiffExp.php) | 497 | 44 | 1922.64 | 5829.84 | 0.33 | 9.40E-22 |
| [LIHC](http://starbase.sysu.edu.cn/panMirDiffExp.php) | 370 | 50 | 2230.23 | 5606.98 | 0.4 | 3.90E-20 |
| [LUAD](http://starbase.sysu.edu.cn/panMirDiffExp.php) | 512 | 20 | 1258.23 | 5235.5 | 0.24 | 1.90E-18 |
| [COAD](http://starbase.sysu.edu.cn/panMirDiffExp.php) | 450 | 8 | 456.87 | 3158.58 | 0.14 | 4.50E-12 |
| [UCEC](http://starbase.sysu.edu.cn/panMirDiffExp.php) | 538 | 33 | 2226.89 | 5006.71 | 0.44 | 6.60E-12 |
| [THCA](http://starbase.sysu.edu.cn/panMirDiffExp.php) | 509 | 58 | 5151.67 | 7597.83 | 0.68 | 8.90E-12 |
| [CHOL](http://starbase.sysu.edu.cn/panMirDiffExp.php) | 36 | 9 | 1121.66 | 5359.93 | 0.21 | 6.80E-11 |
| [KICH](http://starbase.sysu.edu.cn/panMirDiffExp.php) | 65 | 24 | 1271.63 | 2945.24 | 0.43 | 5.10E-10 |
| [LUSC](http://starbase.sysu.edu.cn/panMirDiffExp.php) | 475 | 38 | 1644.09 | 2569.52 | 0.64 | 1.60E-08 |
| [KIRC](http://starbase.sysu.edu.cn/panMirDiffExp.php) | 517 | 71 | 1990.05 | 2158.49 | 0.92 | 0.0053 |
| [STAD](http://starbase.sysu.edu.cn/panMirDiffExp.php) | 372 | 32 | 1397.2 | 1837.24 | 0.76 | 0.0093 |

**Supplementary Table 4. The correlation between the let-7c-5p and IQGAP3 in pan-cancer analysis by the starbase database**

| **Cancer** | **SampleNum** | **Coefficient-R** | **p-value** |
| --- | --- | --- | --- |
| [LUAD](http://starbase.sysu.edu.cn/panMirCoExp.php) | 512 | -0.388 | 7.43E-20 |
| [STAD](http://starbase.sysu.edu.cn/panMirCoExp.php) | 372 | -0.366 | 2.98E-13 |
| [BRCA](http://starbase.sysu.edu.cn/panMirCoExp.php) | 1085 | -0.201 | 2.29E-11 |
| [LIHC](http://starbase.sysu.edu.cn/panMirCoExp.php) | 370 | -0.323 | 1.97E-10 |
| [TGCT](http://starbase.sysu.edu.cn/panMirCoExp.php) | 156 | -0.399 | 2.57E-07 |
| [READ](http://starbase.sysu.edu.cn/panMirCoExp.php) | 161 | -0.369 | 1.45E-06 |
| [ESCA](http://starbase.sysu.edu.cn/panMirCoExp.php) | 162 | -0.366 | 1.62E-06 |
| [COAD](http://starbase.sysu.edu.cn/panMirCoExp.php) | 450 | -0.22 | 2.58E-06 |
| [ACC](http://starbase.sysu.edu.cn/panMirCoExp.php) | 79 | -0.408 | 1.87E-04 |
| [SKCM](http://starbase.sysu.edu.cn/panMirCoExp.php) | 449 | -0.163 | 5.43E-04 |
| [THCA](http://starbase.sysu.edu.cn/panMirCoExp.php) | 509 | -0.149 | 7.28E-04 |
| [KIRC](http://starbase.sysu.edu.cn/panMirCoExp.php) | 517 | -0.147 | 7.71E-04 |
| [PRAD](http://starbase.sysu.edu.cn/panMirCoExp.php) | 495 | -0.148 | 9.67E-04 |
| [CHOL](http://starbase.sysu.edu.cn/panMirCoExp.php) | 36 | -0.505 | 1.68E-03 |
| [KIRP](http://starbase.sysu.edu.cn/panMirCoExp.php) | 289 | -0.162 | 5.63E-03 |
| [UCEC](http://starbase.sysu.edu.cn/panMirCoExp.php) | 538 | -0.119 | 5.85E-03 |
| [LUSC](http://starbase.sysu.edu.cn/panMirCoExp.php) | 475 | -0.113 | 1.40E-02 |
| [SARC](http://starbase.sysu.edu.cn/panMirCoExp.php) | 261 | -0.143 | 2.07E-02 |
| [THYM](http://starbase.sysu.edu.cn/panMirCoExp.php) | 119 | -0.2 | 2.90E-02 |

**Supplementary Table 5. The expression of IQGAP3AR in pan-cancer analysis by the starbase database**

| **Cancer type** | **CancerNum** | **NormalNum** | **CancerExp** | **NormalExp** | **FoldChange** | **pValue** | **FDR** |
| --- | --- | --- | --- | --- | --- | --- | --- |
| [LUSC](http://starbase.sysu.edu.cn/panGeneDiffExp.php) | 501 | 49 | 2.35 | 0.79 | 2.96 | 1.20E-26 | 1.80E-25 |
| [LIHC](http://starbase.sysu.edu.cn/panGeneDiffExp.php) | 374 | 50 | 1.3 | 0.48 | 2.7 | 2.70E-22 | 5.50E-21 |
| [LUAD](http://starbase.sysu.edu.cn/panGeneDiffExp.php) | 526 | 59 | 1.87 | 0.8 | 2.34 | 6.20E-21 | 1.20E-19 |
| [STAD](http://starbase.sysu.edu.cn/panGeneDiffExp.php) | 375 | 32 | 2.18 | 0.8 | 2.73 | 8.20E-20 | 6.20E-18 |
| [PRAD](http://starbase.sysu.edu.cn/panGeneDiffExp.php) | 499 | 52 | 1.53 | 0.89 | 1.72 | 3.40E-16 | 1.20E-14 |
| [COAD](http://starbase.sysu.edu.cn/panGeneDiffExp.php) | 471 | 41 | 2.02 | 0.97 | 2.08 | 3.80E-14 | 4.70E-13 |
| [BLCA](http://starbase.sysu.edu.cn/panGeneDiffExp.php) | 411 | 19 | 2.36 | 0.99 | 2.37 | 4.50E-11 | 1.50E-09 |
| [CHOL](http://starbase.sysu.edu.cn/panGeneDiffExp.php) | 36 | 9 | 2.06 | 0.57 | 3.6 | 4.70E-11 | 7.60E-10 |
| [HNSC](http://starbase.sysu.edu.cn/panGeneDiffExp.php) | 502 | 44 | 1.23 | 0.63 | 1.97 | 9.00E-11 | 1.10E-09 |
| [BRCA](http://starbase.sysu.edu.cn/panGeneDiffExp.php) | 1104 | 113 | 1.55 | 1.07 | 1.44 | 2.00E-10 | 9.70E-10 |
| [UCEC](http://starbase.sysu.edu.cn/panGeneDiffExp.php) | 548 | 35 | 1.62 | 1.04 | 1.55 | 1.50E-06 | 9.80E-06 |
| [ESCA](http://starbase.sysu.edu.cn/panGeneDiffExp.php) | 162 | 11 | 2.86 | 1.6 | 1.78 | 7.00E-05 | 0.0011 |
| [KIRP](http://starbase.sysu.edu.cn/panGeneDiffExp.php) | 289 | 32 | 1.49 | 0.95 | 1.57 | 0.00033 | 0.0015 |

**Supplementary Table 6. The correlation between the let-7c-5p and IQGAP3AR in pan-cancer analysis by the starbase database**

| **Cancer** | **SampleNum** | **Coefficient-R** | **p-value** |
| --- | --- | --- | --- |
| [LUAD](http://starbase.sysu.edu.cn/panMirCoExp.php) | 512 | -0.298 | 6.14E-12 |
| [TGCT](http://starbase.sysu.edu.cn/panMirCoExp.php) | 156 | -0.295 | 1.81E-04 |
| [BLCA](http://starbase.sysu.edu.cn/panMirCoExp.php) | 408 | -0.179 | 2.76E-04 |
| [LGG](http://starbase.sysu.edu.cn/panMirCoExp.php) | 525 | -0.178 | 4.08E-05 |
| [LIHC](http://starbase.sysu.edu.cn/panMirCoExp.php) | 370 | -0.167 | 1.24E-03 |
| [UCS](http://starbase.sysu.edu.cn/panMirCoExp.php) | 56 | -0.161 | 2.37E-01 |
| [BRCA](http://starbase.sysu.edu.cn/panMirCoExp.php) | 1085 | -0.147 | 1.19E-06 |
| [PRAD](http://starbase.sysu.edu.cn/panMirCoExp.php) | 495 | -0.146 | 1.08E-03 |
| [THYM](http://starbase.sysu.edu.cn/panMirCoExp.php) | 119 | -0.144 | 1.18E-01 |
| [ACC](http://starbase.sysu.edu.cn/panMirCoExp.php) | 79 | -0.141 | 2.14E-01 |
| [DLBC](http://starbase.sysu.edu.cn/panMirCoExp.php) | 47 | -0.14 | 3.49E-01 |
| [KIRP](http://starbase.sysu.edu.cn/panMirCoExp.php) | 289 | -0.124 | 3.56E-02 |
| [STAD](http://starbase.sysu.edu.cn/panMirCoExp.php) | 372 | -0.121 | 1.93E-02 |

**Supplementary Table 7. The correlation between the IQGAP3AR and IQGAP3 in pan-cancer analysis by the starbase database**

| **Cancer** | **SampleNum** | **Coefficient-R** | **p-value** |
| --- | --- | --- | --- |
| [DLBC](http://starbase.sysu.edu.cn/panGeneCoExp.php) | 48 | 0.651 | 5.53E-07 |
| [TGCT](http://starbase.sysu.edu.cn/panGeneCoExp.php) | 156 | 0.627 | 2.05E-18 |
| [LUSC](http://starbase.sysu.edu.cn/panGeneCoExp.php) | 501 | 0.458 | 2.61E-27 |
| [THYM](http://starbase.sysu.edu.cn/panGeneCoExp.php) | 119 | 0.429 | 1.14E-06 |
| KIRC | 535 | 0.411 | 3.34E-23 |
| [ACC](http://starbase.sysu.edu.cn/panGeneCoExp.php) | 79 | 0.408 | 1.91E-04 |
| [LUAD](http://starbase.sysu.edu.cn/panGeneCoExp.php) | 526 | 0.386 | 4.39E-20 |
| [PRAD](http://starbase.sysu.edu.cn/panGeneCoExp.php) | 499 | 0.382 | 9.69E-19 |
| [LGG](http://starbase.sysu.edu.cn/panGeneCoExp.php) | 529 | 0.38 | 1.21E-19 |
| [STAD](http://starbase.sysu.edu.cn/panGeneCoExp.php) | 375 | 0.367 | 2.26E-13 |
| [KIRP](http://starbase.sysu.edu.cn/panGeneCoExp.php) | 289 | 0.348 | 1.15E-09 |
| [CESC](http://starbase.sysu.edu.cn/panGeneCoExp.php) | 306 | 0.344 | 6.10E-10 |
| [PAAD](http://starbase.sysu.edu.cn/panGeneCoExp.php) | 178 | 0.325 | 9.61E-06 |
| [BRCA](http://starbase.sysu.edu.cn/panGeneCoExp.php) | 1104 | 0.316 | 5.76E-27 |
| [READ](http://starbase.sysu.edu.cn/panGeneCoExp.php) | 167 | 0.309 | 4.74E-05 |
| [UCEC](http://starbase.sysu.edu.cn/panGeneCoExp.php) | 548 | 0.278 | 3.74E-11 |
| [PCPG](http://starbase.sysu.edu.cn/panGeneCoExp.php) | 183 | 0.267 | 2.60E-04 |
| [HNSC](http://starbase.sysu.edu.cn/panGeneCoExp.php) | 502 | 0.258 | 4.20E-09 |
| [KICH](http://starbase.sysu.edu.cn/panGeneCoExp.php) | 65 | 0.246 | 4.87E-02 |
| [COAD](http://starbase.sysu.edu.cn/panGeneCoExp.php) | 471 | 0.241 | 1.23E-07 |
| [ESCA](http://starbase.sysu.edu.cn/panGeneCoExp.php) | 162 | 0.236 | 2.53E-03 |
| [SARC](http://starbase.sysu.edu.cn/panGeneCoExp.php) | 263 | 0.222 | 2.78E-04 |
| [SKCM](http://starbase.sysu.edu.cn/panGeneCoExp.php) | 471 | 0.194 | 2.20E-05 |
| [CHOL](http://starbase.sysu.edu.cn/panGeneCoExp.php) | 36 | 0.187 | 2.74E-01 |
| [OV](http://starbase.sysu.edu.cn/panGeneCoExp.php) | 379 | 0.168 | 1.05E-03 |
| [BLCA](http://starbase.sysu.edu.cn/panGeneCoExp.php) | 411 | 0.165 | 7.57E-04 |
| [MESO](http://starbase.sysu.edu.cn/panGeneCoExp.php) | 86 | 0.163 | 1.33E-01 |
| [LIHC](http://starbase.sysu.edu.cn/panGeneCoExp.php) | 374 | 0.143 | 5.52E-03 |
| [THCA](http://starbase.sysu.edu.cn/panGeneCoExp.php) | 510 | 0.108 | 1.43E-02 |

**Supplementary Table 8. The correlation between the IQGAP3 expression and drug sensitivity in diverse cancer cells lines analysis by the GDSC database**

| symbol | drug | cor | fdr |
| --- | --- | --- | --- |
| IQGAP3 | TPCA-1 | 0.153424 | 9.18E-06 |
| IQGAP3 | Vorinostat | 0.15331 | 2.07E-05 |
| IQGAP3 | Methotrexate | 0.143143 | 7.72E-05 |
| IQGAP3 | PHA-793887 | 0.126388 | 0.000279 |
| IQGAP3 | PIK-93 | 0.126585 | 0.000285 |
| IQGAP3 | XMD13-2 | 0.125644 | 0.000363 |
| IQGAP3 | BHG712 | 0.121255 | 0.000624 |
| IQGAP3 | AR-42 | 0.119278 | 0.000751 |
| IQGAP3 | CUDC-101 | 0.119644 | 0.000893 |
| IQGAP3 | Ispinesib Mesylate | 0.118217 | 0.000947 |
| IQGAP3 | SNX-2112 | 0.117472 | 0.00099 |
| IQGAP3 | OSI-027 | 0.11653 | 0.00106 |
| IQGAP3 | Vinblastine | 0.130314 | 0.001081 |
| IQGAP3 | PI-103 | 0.11509 | 0.001222 |
| IQGAP3 | CEP-701 | 0.124121 | 0.001248 |
| IQGAP3 | KIN001-260 | 0.114958 | 0.001257 |
| IQGAP3 | I-BET-762 | 0.111259 | 0.00139 |
| IQGAP3 | CAY10603 | 0.107329 | 0.00251 |
| IQGAP3 | CP466722 | 0.107009 | 0.002553 |
| IQGAP3 | AT-7519 | 0.10463 | 0.003152 |
| IQGAP3 | AZD8055 | 0.111845 | 0.003548 |
| IQGAP3 | Tubastatin A | 0.103315 | 0.003588 |

**Supplementary Table 9. The correlation between the IQGAP3 expression and drug sensitivity in diverse cancer cells lines analysis by the CTRP database**

| symbol | drug | cor | fdr |
| --- | --- | --- | --- |
| IQGAP3 | SB-225002 | 0.240618 | 3.55E-11 |
| IQGAP3 | LY-2183240 | 0.241652 | 4.53E-11 |
| IQGAP3 | apicidin | 0.228082 | 4.74E-10 |
| IQGAP3 | cytarabine hydrochloride | 0.228543 | 4.75E-10 |
| IQGAP3 | doxorubicin | 0.220865 | 1.28E-09 |
| IQGAP3 | CR-1-31B | 0.221029 | 1.54E-09 |
| IQGAP3 | vincristine | 0.214242 | 2.78E-09 |
| IQGAP3 | panobinostat | 0.21695 | 3.62E-09 |
| IQGAP3 | etoposide | 0.215136 | 5.86E-09 |
| IQGAP3 | BI-2536 | 0.211526 | 6.16E-09 |
| IQGAP3 | I-BET151 | 0.21346 | 6.17E-09 |
| IQGAP3 | obatoclax | 0.214338 | 9.12E-09 |
| IQGAP3 | tipifarnib-P1 | 0.21734 | 1.34E-08 |
| IQGAP3 | KX2-391 | 0.211997 | 1.38E-08 |
